# The genome of Rhizobiales bacteria in predatory ants reveals urease gene functions but no genes for nitrogen fixation

**DOI:** 10.1038/srep39197

**Published:** 2016-12-15

**Authors:** Minna-Maria Neuvonen, Daniel Tamarit, Kristina Näslund, Juergen Liebig, Heike Feldhaar, Nancy A. Moran, Lionel Guy, Siv G. E. Andersson

**Affiliations:** 1 Department of Molecular Evolution, Cell and Molecular Biology, Science for Life Laboratory, Biomedical Centre, Uppsala University, SE-752 36 Uppsala, Sweden; 2 School of Life Sciences, Arizona State University, Tempe, AZ, 85287, USA; 3 Animal Population Ecology, Department of Animal Ecology I, Bayreuth Center of Ecology and Environmental Research (BayCEER), University of Bayreuth, D-95440, Bayreuth, Germany; 4 Department of Integrative Biology, University of Texas, Austin, Texas, USA; 5 Department of Medical Biochemistry and Microbiology, Uppsala University, Biomedical Centre, SE-751 23 Uppsala, Sweden

## Abstract

Gut-associated microbiota of ants include Rhizobiales bacteria with affiliation to the genus *Bartonella*. These bacteria may enable the ants to fix atmospheric nitrogen, but no genomes have been sequenced yet to test the hypothesis. Sequence reads from a member of the Rhizobiales were identified in the data collected in a genome project of the ant *Harpegnathos saltator*. We present an analysis of the closed 1.86 Mb genome of the ant-associated bacterium, for which we suggest the species name *Candidatus* Tokpelaia hoelldoblerii. A phylogenetic analysis reveals a relationship to *Bartonella* and *Brucella*, which infect mammals. Novel gene acquisitions include a gene for a putative extracellular protein of more than 6,000 amino acids secreted by the type I secretion system, which may be involved in attachment to the gut epithelium. No genes for nitrogen fixation could be identified, but genes for a multi-subunit urease protein complex are present in the genome. The urease genes are also present in *Brucella,* which has a fecal-oral transmission pathway, but not in *Bartonella*, which use blood-borne transmission pathways. We hypothesize that the gain and loss of the urease function is related to transmission strategies and lifestyle changes in the host-associated members of the Rhizobiales.

Nutritional bacterial symbionts are hypothesized to enable the use of nitrogen-poor diets by a range of arboreal ants. *Blochmannia* is the only internally housed bacterial symbiont with direct evidence for nutrient provisioning in ants. This intracellular endosymbiont enables carpenter ants (genus *Camponotus*) to thrive on food sources that are rich in carbohydrates but poor in proteins, such as honeydew produced by aphids. *Blochmannia* grows in bacteriocytes in the midgut tissue in carpenter ants and contributes to the nutritional cycle through nitrogen recycling and upgrading of nonessential amino acids^1^,^2^,^3^. Likewise, species of tropical arboreal ants (genus *Tetraponera*), which feed on nitrogen-poor homopteran exudates, contain gut symbionts related to nitrogen-fixing root-nodule bacteria, which was used to hypothesize a nutritional function via nitrogen fixation^4^. These ants contain a specialized gut pocket that is enclosed by the trachaea, suggesting that aerial nitrogen may potentially come close enough to the gut to be fixed^4^. Consistently, the *nifH* gene for the dinitrogenase complex was identified in the microbiome of *Tetraponera*^5^, supporting the hypothesis that this capability was the basis for a mutualistic interaction with ants^6^.

Broad surveys of the taxonomic composition of the gut microbiomes of ants have revealed several ant-specific bacterial lineages, consistent with symbiotic relationships^4^,^6^,^7^. The most prevalent such lineage is a clade that belongs to the order Rhizobiales, which is present in about 5% of the surveyed ants^6^,^7^. These bacteria are highly represented in ants at the herbivorous end of the trophic scale, leading to the hypothesis that they are nutritional symbionts^6^,^7^. The distribution profiles indicate at least five independent acquisitions of the Rhizobiales symbionts, which have allowed several ant lineages to colonize ecological niches that would otherwise not be accessible. The symbionts have thereby played a key role in ant diversification^6^.

The order Rhizobiales contains bacterial species that are agriculturally important and able to fix atmospheric nitrogen and to recycle nitrogenous waste products such as urea into ammonium for incorporation into amino acids. The closest bacterial relatives of the ant-associated Rhizobiales among cultivated strains with sequenced genomes belong to the genus *Bartonella*^8^,^9^ and lack the ability to fix nitrogen or recycle urea. These bacteria infect endothelial cells and erythrocytes in mammals, and are transmitted between hosts by blood-sucking arthropods^10^. The infections are asymptomatic in most animals, although a few *Bartonella* species are recognized human pathogens. However, despite the potential importance of the microbiome for ant ecology, no bacterial isolates are available and no genomic studies of the ant-associated members of the Rhizobiales clade have been performed.

The genomes of the ants *Camponotus floridanus* and *Harpegnathos saltator* have been sequenced and analyzed^11^. These two ant species have contrasting social behaviors and diets. The *Camponotus* ants, also called carpenter ants, are adapted to carbohydrate rich diets and live in large and well-organized colonies with a high degree of task specialization and territoriality. In contrast, ants of the genus *Harpegnathos*, also called Indian jumping ants, have small colonies and are solitary hunters with basic task specialization and low territoriality. These ants can jump several centimeters and prey on small, living arthropods that they capture with their elongated mandibles. Thus, they have access to a diet that is rich in proteins. The assembly of the raw sequence reads obtained from the *H. saltator* genome project contained one scaffold with sequence similarity to the ant-specific clade of the Rhizobiales^11^. Although these bacteria are mostly associated with herbivory in ants, they have also in a few cases been identified in predatory ants of the genus *Pheidole*^6^ and also in omnivorous giant tropical ants of the genus *Paraponera*^12^ However, no complete genome sequences are as yet available for the ant-specific group of Rhizobiales bacteria, nor is it known what effects these bacteria have on the health and lifestyle of the ants.

Here, we have used the bacterial sequence scaffold obtained in the genome project of *H. saltator* to close the bacterial genome with the aid of PCR reactions targeted to regions that contain gaps. We show that the ant-associated bacteria diverged prior to the diversification of the *Bartonella spp.* and suggest that it represents a distinct species and genus. We present a broad genomic comparison of gene functions with those identified in the genera *Brucella* and *Bartonella*, which infect mammals. We find that the ant-associated bacterial species of the Rhizobiales clade resembles *Bartonella* and *Brucella* in their lack of capacity to fix nitrogen, but like *Brucella*, and in contrast to *Bartonella*, contain genes for the urease protein complex. We discuss the possible implications of these findings for ant lifestyles and the pathways involved in the emergence of vector-borne *Bartonella* pathogens.

## Results

### Genome features

**Figure 1:**
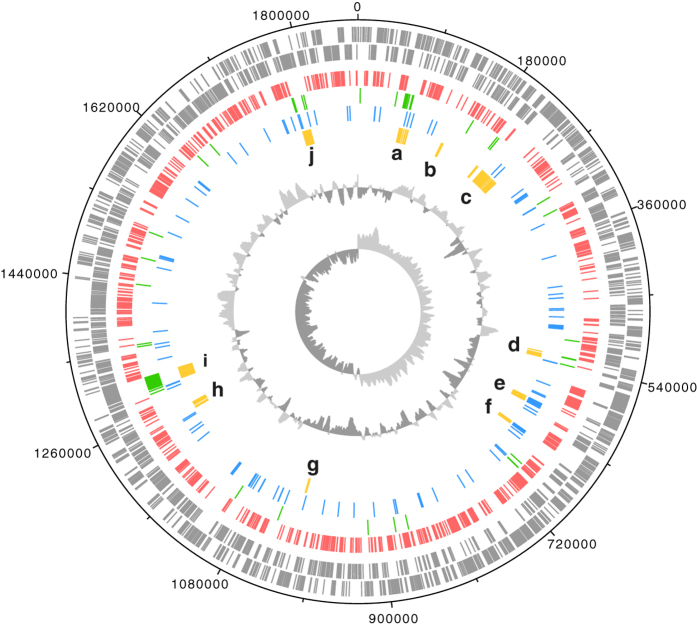
Circular representation of the Bhsal genome. Features from the outer circle to the center are: genes on the forward strand, genes on the reverse strand, single-copy orthologs from all the 17 surveyed genomes (red), genes uniquely present in Bhsal and a few other species (green), singletons in Bhsal (blue) and genes coding for proteins of special interest (yellow) such as: a, GTA-like phage; b, *Bartonella* adhesin (BadA); c, type III secretion system (T3SS); d, urease; e, CRISPR-cas type I-C; f, CRISPR-cas type II-C; g, BadA; h, autotransporters; i, putative extracellular protein secreted by the type I secretion system; j, filamentous hemagglutinin (FHA). The two innermost circles show the GC-bias and the GC-skew. The figure was obtained with dnaplotter^51^, and edited with Adobe Illustrator.

The sequence reads of putative bacterial origin identified in the *H. saltator* genome data were assembled into a single circular scaffold consisting of about 120 contigs^11^, with a coverage of about 140X, whereas the ant genome coverage was 104X. We extracted bacterial DNA from *H. saltator* and performed PCR reactions on the genomic DNA to bridge the gaps in the scaffold. The sequences obtained from the PCR products were added to the assembly and the resulting genome size was estimated to be 1.86 Mb (Fig. 1). The high coverage of the bacterial scaffolds excludes the hypothesis that they represent a low-level contaminant. We also consider it unlikely that Bhsal represents an incidental infection since it is consistently isolated from the same laboratory-kept colonies. Below, we refer to the bacterial species from which the genome was obtained with the abbreviation Bhsal (*Bartonella* in *H. saltator*).

The characteristic GC-skew pattern (central circle in Fig. 1) provided strong support for the assembly, and the two shifts in the direction of the bias at equidistant positions in the genome were used to identify the origin and terminus of replication. The presence of a *dif-*site at one of these positions confirmed the predicted terminus of replication^13^. Although the *dnaA* gene was not located in the vicinity of the predicted origin of replication, and we were unable to identify *dnaA* boxes, the gene segment *gidAB*-*parAB* was located near the predicted origin, as found in other Alpha-proteobacteria^14^, and was surrounded by sequences identical to consensus *parS* palindromes^15^, which also accumulate around the origin of replication.

In total, 1688 protein coding sequences were predicted with an average size of 992 bp. In addition, the genome contained two complete rRNA operons and 46 tRNA genes. It also contained pseudogenes for the mismatch repair protein MutS, the repair-related helicase UvrD, the homoserine dehydrogenase, a catalase, a copper homeostasis protein and a putative methyltransferase. We identified genes for the *Bartonella* adhesin (BadA), a flagellar type III secretion system (T3SS), filamentous hemagglutinin (FHA), and the *Bartonella* gene transfer agent (BaGTA), which are thought to have played an important role in the evolution of the canonical *Bartonella* species^9^. However, unlike the clustering of genes for secretion systems in the canonical *Bartonella* genomes, these genes were not located in a specific segment of the genome (Fig. 1).

### Phylogenetic analyses

**Figure 2:**
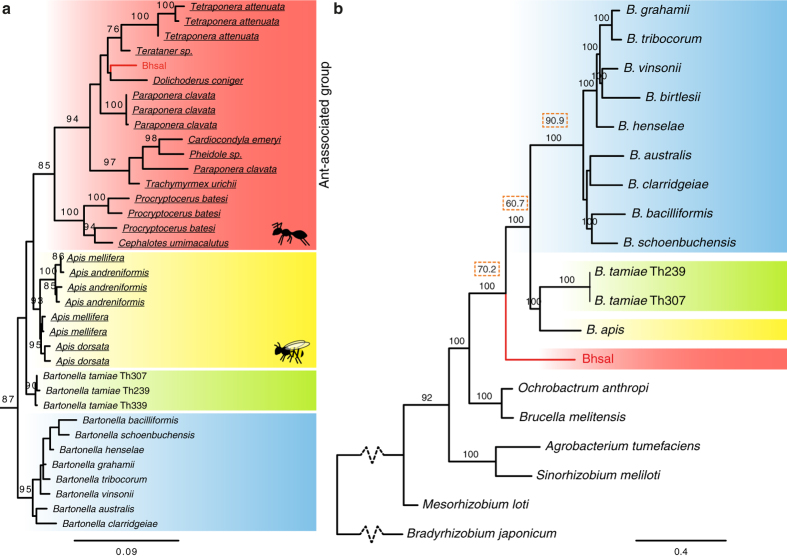
Phylogenetic placement of Bhsal. The phylogenies were based on (**a**) the 16 S rRNA gene and (**b**) a concatenated protein alignment. Colors represent the ant-associated sequence (red), the bee-associated sequences (yellow), the *B. tamiae* lineage (lime), and the clade formed by the canonical *Bartonella* species (blue). Ant and bee cliparts represent groups of sequences obtained from ant and bee samples, respectively. In (**a**), only the clade containing the *Bartonella* and the sequences obtained from ant and bee samples are shown; the complete tree is shown in Supplementary Fig. S1. In (**b**), dashed squares above key branches represent the percentage of single-gene trees that include those branches with high support (>70%), out of the total of 630 single-copy panorthologs. Both trees were inferred with the maximum likelihood method. Only bootstrap values higher than 80% are shown. The figure was drawn with Figtree (Andrew Rambaut, available on the author’s website: http://tree.bio.ed.ac.uk/software/figtree/), and edited with Adobe Illustrator.

A maximum likelihood phylogeny inferred from the 16 S rRNA gene sequences showed that Bhsal belongs to a genetically diverse clade (94% bootstrap support) of bacterial strains isolated from herbivorous ants (*Tetraponera attenuata* and *Dolichoderus coniger*), omnivorous ants (*Paraponera clavata*) as well as from predatory ants (*Pheidole* sp.) (Fig. 2; Supplementary Fig. S1). This monophyletic group is a sister-clade to another ant-associated clade, which contains 16 S rRNA sequences amplified from ants of the genera *Cephalotes* and *Procryptocerus* (100% bootstrap support). The separation of the 16 S rRNA gene sequences amplifyied from ants into several distinct clades has also been noted previously^6^,^7^. Pairwise 16 S rRNA sequence comparisons indicated at the most 97.5% sequence identity of Bhsal to the most closely related bacteria isolated from ants, and between 94.1% and 95.5% sequence identity of Bhsal to the other *Bartonella* species (Supplementary Table S1).

A related insect-associated clade is the Alpha-1 group of bacteria present in honeybees of the genus *Apis*, with the assigned species designation *Bartonella apis*^16^. Consistent with previous studies^9^, the coherence of the canonical *Bartonella* species was supported by 95% of the bootstraps, while *B. tamiae* clustered outside this group as observed previously^17^,^18^. However, the order in which *B. tamiae*, the honeybee- and the two ant-associated clades diverged from each other could not be resolved in the 16 S rRNA phylogeny with significant statistical support.

To obtain a better resolution of the diversification patterns between the three clades of arthropod-associated *Bartonella*-like strains, we turned to protein sequences. We clustered the proteins encoded by the Bhsal genome and a representative set of *Bartonella* genomes using OrthoMCL (requiring E < 10^−5^ and an alignment of >50% of the protein lengths) (Supplementary Table S2). We also included the protein sequences encoded by an assembled honeybee gut metagenome dataset from *B. apis*^19^, as well as the proteomes of six other Rhizobiales species, here used as outgroups. The clustering identified 629 (with *B. apis*) and 647 (without *B. apis*) protein families that contained proteins encoded by single copy genes in each genome. However, due to the high coverage of reads and the sample diversity of the gut metagenome dataset, *B. apis* were often represented by 4–5 protein copies of varying lengths. Manual inspection of single protein phylogenies confirmed that the copies were monophyletic (or in 8 cases paraphyletic with the inclusion of *B. tamiae*). Thus, the selection of a specific sequence would not affect the tree topology although the protein copies encoded by the metagenome may represent different strains of *B. apis*. To increase the statistical power, we selected the longest metagenome sequence in each family for further analysis.

A single maximum likelihood phylogenetic tree was inferred from a concatenated alignment of all 629 proteins (Fig. 2). The branching pattern within the main *Bartonella* clade was similar to the previously published tree topology^9^, although we made no attempt in this study to further resolve internal nodes with low support. Importantly, the tree topology suggested with 100% bootstrap support that Bhsal diverged prior to the sister groups represented by *B. tamiae* and *B. apis*, all three of which subtended the canonical *Bartonella spp*. Consistently, more than 60% of the single protein trees suggested that Bhsal diverged prior to *B. tamiae* and *B. apis* with more than 70% bootstrap support (Fig. 2).

However, we were concerned that the GC content of the Bhsal genome (54%) is substantially higher than the genomic GC content of the other *Bartonella* species (37–42%), and thus more similar to the genomic GC contents of the outgroup genera *Ochrobactrum* and *Brucella* than the others (54–57%). Such a bias in the dataset could lead to a situation in which species with similar GC contents could be artificially attracted to each other during tree reconstruction, leaving out members of otherwise monophyletic groups. To test for artifacts caused by nucleotide compositional biases, we examined the topologies of single protein trees inferred from alignments of the 50 genes that differed the least in GC content at the first two codon positions in Bhsal and *Bartonella* in the 647 gene dataset that excluded the *B. apis* metagenome (Supplementary Table S3). In 39 phylogenies, the canonical *Bartonella spp.* formed a monophyletic group (>70% bootstrap support), which was subtended by *B. tamiae* in 26 cases. No single gene tree indicated a close relationship of Bhsal to any of the other *Bartonella* species with strong support, suggesting that its deep divergence is not an artifact of its relatively higher GC-content. The preferred placement of Bhsal was the one represented by the concatenated tree, as observed in 14 trees with >70% bootstrap support. Four single protein trees supported this placement with 100% bootstrap support, including trees inferred from the 601 amino acids long RecJ protein and a hypothetical protein of 1554 amino acids with an apolipoprotein domain, which are encoded by contiguous genes. The inclusion of the *B. apis* metagenome reads in the latter phylogenies yielded the same diversification pattern of Bhsal, *B. apis, B. tamiae* and the canonical *Bartonella* species as in the concatenated tree with over 97% bootstrap at each node. For these reasons, we favor the tree topology shown in Fig. 2.

This phylogeny shows that the currently identified blood-borne, vector-transmitted *Bartonella* species are paraphyletic: two independent clades are adapted to arthropods only (Bhsal and *B. apis*), and two clades are adapted to living in the blood of mammals and transmitted via ectoparasites (*B. tamiae* and the canonical *Bartonella* species). This suggests various scenarios, among which at least two are equally parsimonious: (i) that the ability to infect the blood of mammals through blood-sucking arthropods occurred twice, or (ii) that the ancestor of *B. apis* could infect the blood of mammals but lost this ability.

### Adaptive genomic changes in the ant gut bacteria

**Figure 3:**
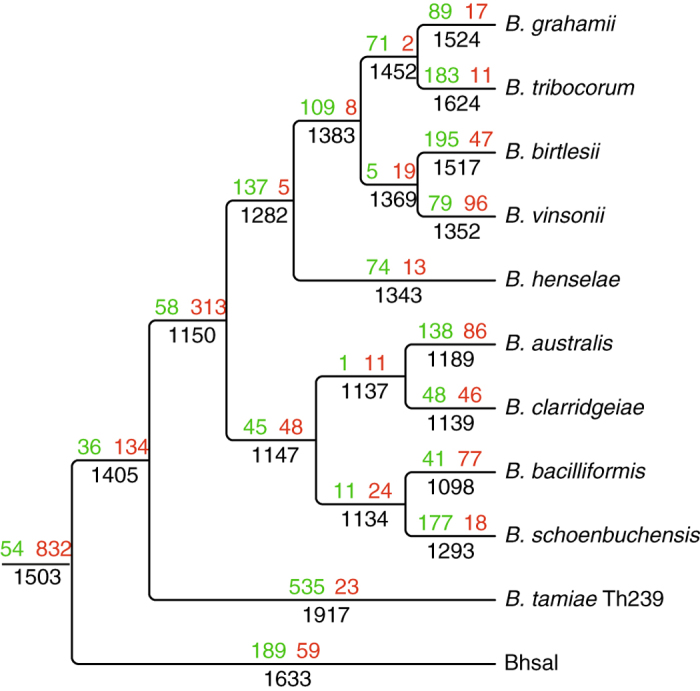
Gene flux analysis. Gains and losses of protein families were mapped onto the reference phylogeny of the Bhsal, *B. tamiae* and the canonical *Bartonella* species shown in Supplementary Fig. S2a. Above each branch, gains of protein families are shown in green, while losses are shown in red. Beneath each branch, the total number of protein families is shown. Only the ingroup is shown; the full tree and the full set of family gains and losses are shown in Supplementary Fig. S2b. The figure was drawn using custom perl and bash scripts, and edited with Adobe Illustrator.

With a size of 1.86 Mb, the Bhsal genome is within the size range of the other *Bartonella* genomes (1.4–2.6 Mb), and thus substantially smaller than the genomes of the sister genera *Ochrobactrum* and *Brucella* species (>3 Mb, Supplementary Table S2). To study the transition from commensal gut bacteria of ants and other insects to blood-borne mammalian pathogens, we inferred the flux of genes at the ancestral nodes. Gains and losses of protein families along the reference *Bartonella* tree were inferred by maximum parsimony (Fig. 3, Supplementary Fig. S2). We included *B. tamiae* in the analysis but excluded the Alpha-1 metagenome dataset from *B. apis* due to its incomplete status.

A previous gene flux analysis with fewer taxa indicated a loss of about 1,500 protein families and a gain of about 100 protein families on the branch to *Bartonella*^9^. Consistently, our gene flux analysis confirmed that the loss of genes has been massive, while the acquisition of genes has been a much slower process, except in *B. tamiae* in which 535 protein families were acquired. An alternative scenario is that the protein families solely identified in *B. tamiae* were present in the ancestor, and lost independently in Bhsal and the canonical *Bartonella* spp.

Also consistent with previous studies, our analysis indicated that the acquisition of genes for secretion systems has been instrumental for the invasion into new host species and the explosive radiation of the *Bartonella* genus^9^. In total, 54 protein families were acquired at the node representing the ancestor of Bhsal and *Bartonella* (Supplementary Table S4). These included genes for filamentous hemagglutinin and HecB, which have been lost and horizontally acquired many times in the individual *Bartonella* species^9^. Another 58 protein families were acquired at the node to the canonical *Bartonella spp.* (Supplementary Table S5). Prime among these were the *virB* genes for type IV secretion systems, which are present in a subset of *Bartonella* species and have been shown experimentally to mediate adhesion to endothelial cells^20^.

**Figure 4:**
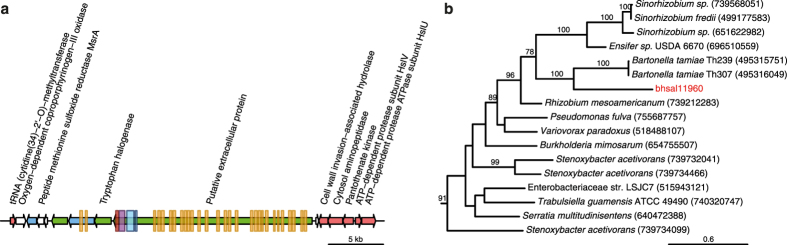
Domain structure of a putative extracellular protein secreted by the type I secretion system. (**a**) Gene order structure of the segment containing genes for a giant protein (putative extracellular protein) and a tryptophan halogenase. Arrows represent genes, and colors represent orthologs (red), genes uniquely present in Bhsal and a few other species (green), singletons in Bhsal (blue). Rectangles represent hits to Bacterial Ig-like domain (group 3) repeat IPR022038 (orange), SCOP family integrin alpha N-terminal domain SSF69318 (blue), serralysin-like metalloprotease C-terminal domain IPR011049 (purple) and the TIGRFam domain Type I secretion C-terminal target domain IPR019960 (red). (**b**) Phylogeny of tryptophan halogenase, where GI numbers are shown in parentheses, and only bootstrap values above 75 are shown. The figure was generated with genoplotR^52^, and edited with Adobe Illustrator.

To search for traits that might provide clues about the lifestyle of Bhsal, we inspected the 189 protein families identified as gained (Supplementary Table S6). Of these, 79 protein families have annotated functions, the most notable of which is a protein that is 6,177 amino acids in length and annotated as a “putative extracellular giant protein” (Fig. 4). This protein contains a series of repeated domains, which are similar to bacterial Ig-like (BIg) repeat domains of class BIg3. The C-terminus of the protein yielded a superfamily hit to the C-terminal domain of a serralysin-like metalloprotease (positions 5,600–5,840) (E < 10^−12^), and a TIGRFAM hit to a C-terminal domain of proteins secreted by the type 1 secretion pathway (positions 6,099–6,176) (E < 10^−10^). Based on the domain structure, we predict that the giant protein is secreted through the cell membrane with the help of a type 1 secretion system (T1SS). Consistently, three genes for a T1SS were identified immediately next to the inferred origin of replication in the Bhsal genome.

Type I secretion systems are widespread in bacteria and consist of three proteins that span the cellular membrane; an ATP-binding protein of the ABC class, a membrane fusion protein (MFP), and an outer membrane protein (OMP) of the TolC class^21^. The proteins secreted by this system have been identified as hydrolases, toxins or adhesion molecules^21^. Several of these proteins contain glycine-rich repeats that bind calcium ions. For example, the SiiE protein of *Salmonella enterica* is 5,559 amino acids long and contains 53 BIg3_4 domains in addition to the T1SS recognition domain^22^. The SiiE protein has been shown to interact with more than 100 calcium ions and to mediate adhesion to polarized epithelial cells^23^. Based on the crystal structure of one of the BIg domains, it is suggested that the calcium ions provide a cross-link between the LPS molecule of the bacterial cell, whereas the surface exposed loops of the protein interact with the host cell carbohydrates or phospholipids^22^. Since the extracellular giant protein in Bhsal has a very similar domain organization as the SiiE protein, we hypothesize that it mediates attachment to the lining of the ant gut.

Genes for such a T1SS as well as a gene for a protein with IG-like domain repeats were also identified in the *B. tamiae* genome, but not in the genomes of the canonical *Bartonella* species. However, the sequence similarity between this protein and the giant extracellular protein in Bhsal was only observed over a short segment and the *B. tamiae* protein contains additional domains not present in the Bhsal protein. Located immediately downstream of the gene for the putative extracellular protein in Bhsal was a gene for tryptophan halogenase, which is an enzyme that incorporates halogens (chlorine, bromide, etc.) into organic molecules, such as antibiotics that might serve a putative role in the competition against other bacteria^24^. A phylogeny of tryptophan halogenase showed a sister relationship for Bhsal and *B. tamiae*, indicating that they may have been acquired in the common ancestor and lost in the canonical *Bartonella* species (Fig. 4B). Moreover, the organization of these genes is conserved in Bhsal and *B. tamiae*. Taken together, this suggests that *B. tamiae* represents an intermediate strain that has retained certain characteristics with Bhsal despite its association with humans.

Many transporters and mobile elements were identified as gained in Bhsal, including also two cassettes for CRISPR-*cas* systems (Clustered Regularly Interspaced Short Palindromic Repeats, and CRISPR-associated proteins). Neither is present in any member of the canonical *Bartonella spp.*, nor are they present in the Rhizobiales genomes used as outgroup in this study. The first CRISPR-*cas* cassette spans a region of ~10 kb and is classified as type I-C^25^ encompassing the *cas3* gene and additional *cas5, cas8c*/*csd1, cas7*/*csd2, cas4, cas1* and *cas2*. It is followed by a 33 repeats-long spacer array (Supplementary Fig. S3). The second CRISPR-cas region is 5 kb long and is classified as type II-C bearing the signature *cas9* gene followed by *cas1* and *cas2* and the 9 repeats-long spacer array. The two CRISPR cassettes flank a region with 15 genes coding for NADH dehydrogenases.

A blastn search of spacer26 in Bhsal CRISPR type I-C region to the NCBI’s nonredudant nucleotide database yielded only a hit to the bacteriophage Mu in *Rhodobacter capsulatus* with only 2 mismatches (E = 2E-04), whereas a search to a local nucleotide sequence database consisting of *Bartonella* genomes (Supplementary Table S2) yielded no significant hits (E > 0.01). Nor did the spacer sequences yield any significant hits to known mobile elements^26^.

### No genes for nitrogen fixation

A still unresolved question is the nature of the interaction between Bhsal and its host. One hypothesis is that the ant symbionts within Rhizobiales enable their hosts to fix atmospheric nitrogen^6^, thereby enabling the switch to herbivorous lifestyles dependent on nitrogen-poor diets. Indeed, the *nifH* gene was detected by PCR in the gut microbiome of *Tetraponera* and *Dolichoderus*, both relatively herbivorous ants known to host relatives of Bhsal^5^. However, the Bhsal genome lacks genes for nitrogen fixation, so if this species is representative of other ant-associated bacteria in the same clade, this function may not be the basis for herbivory in ants.

### Biosynthetic capabilities

Another hypothesis is that Bhsal produces amino acids for the ant, a capability that is found in several other insect symbionts, such as *Blattabacterium* in cockroaches^27^ and *Blochmannia* in carpenter ants^3^. Such a role is also possible for *B. apis* that live on diets that are rich in carbohydrates, but poor in proteins. We identified genes for the biosynthesis of all essential amino acids in Bhsal although the pathways for methionine and phenylalanine were predicted to lack one or two steps, and only one gene coding for a protein involved in histidine biosynthesis has been kept, raising the possibly that these amino acids are supplied by the ant. Indeed, *H. saltator* is a carnivore and as such is not normally expected to need any extra supply of amino acids. Consistent with the utilization of host proteins, the acquired functions in Bhsal include 17 protein families associated with protein degradation and amino acid transport, such as hydrolases, peptidases, aminotransferases, dehydrogenases and ABC transporters.

Yet another hypothesis is that Bhsal produces vitamins for the host. Many insect endosymbionts like *Baumannia* and *Blochmannia* supplement their hosts with vitamins. To address the possibility that Bhsal is a vitamin supplier we searched for vitamin biosynthetic pathways. Bhsal has a complete set of genes (*ribABC/EDFH*), to synthesize riboflavin (vitamin B2), which is essential for the synthesis of flavin adenine dinucleotide (FAD) and flavin mononucleotide (FMN) that are important cofactors in many metabolic processes^28^. This pathway is encoded by *ribABCDH* genes in *E. coli* as well as in *Blochmannia* of *Camponotus*^29^, which are thought to provide the ant host with vitamin B2. As animals lack the pathway to vitamin B2 and since Bhsal has retained the full pathway, it is possible that *H. saltator* obtains this vitamin from Bhsal. On the other hand, Bhsal contains only a partial pathway for tetrahydrofolate and biotin (vitamin B7), and the gene flux analyses revealed losses of genes for the biosynthesis of vitamin B1 and vitamin B6, perhaps indicating that these vitamins are obtained from the ant diet.

Thus, the most likely scenario is that Bhsal belongs to a commensal bacterial population that takes advantage of the rich food resources present in the ant gut. Consistently, the inferred loss of 59 protein families on the branch to Bhsal has mostly affected biosynthetic pathways, including the loss of genes for shikimate dehydrogenase involved in the pathway to phenylalanine, tryptophan and tyrosine and pseudogenization of the gene for homoserine Dehydrogenase involved in the pathway leading to threonine, methionine and isoleucine (Supplementary Table S7). Additionally, the *carA* and *carB* genes catalysing the conversion of ammonia to carbamoyl phosphate have been lost, as have also genes for the biosynthesis of coenzyme A, acetyl CoA, thiamine (vitamin B1) and pyridoxal phosphate (vitamin B6). (Supplementary Table S7). Furthermore, the losses included all genes in the Entner-Doudoroff pathway. Another cellular function that seems suppressed is that of DNA repair functions including the loss of genes for DNA polymerase I and the epsilon subunit of the DNA polymerase III, and the pseudogenizations of the genes for MutS and UvrD.

However, the reduction in the biosynthetic repertoire of genes does not exclude the possibility that Bhsal provides nutrients to the ant under exceptional circumstances in which access to prey is limited. For example, drought, lack of prey and floods are environmental factors that might cause nitrogen limitation and thereby lead to a dependency on the gut microbiome. Interestingly, in the omnivorous ant *Paraponera clavata*, the prevalence of *Bartonella*-like bacteria increased when the diet was supplemented with carbohydrates^12^. A long-term shift in diet may thus induce the evolution of an obligate nutritional symbiotic relationship with commensal gut bacteria that normally exploit the gut as a nutrient-rich growth habitat. Comparative studies with *Bartonella*-like bacteria isolated from herbivorous ants may provide clues to the roles that these bacteria have played for the adaptation of ants to new habitats, and thereby to the diversification of ants.

### Switches from commensal gut bacteria to blood-transmitted lifestyles

Since the recycling of nitrogenous waste products, such as urea, into essential amino acids is a key function in the symbioses of *Blochmannia* with carpenter ants, we inspected whether Bhsal and the *Bartonella* species also have this capability. While insects are considered as being predominantly uricotelic animals, most insects seem to be capable of urea production and excretion. In some insects such as the carnivorous dipteran *Sarcophage ruficornis*, enzymes of the urea cycle were shown to be active in the tissue^30^. The genome of *H. saltator* encodes a partial urea cycle that allows the production of urea from arginine^11^. The hydrolysis of urea is catalyzed by urease, yielding ammonium and carbon dioxide. The ammonium is then converted into glutamine by the glutamine synthetase encoded by the *glnA* gene. The bacterial urease is a multi-subunit protein complex, which consists of three structural subunits and several accessory proteins. We identified a cluster of genes coding for all subunits of the urease in the Bhsal genome. Located immediately upstream of these genes was the *glnA* gene for glutamine synthetase, and a contiguous gene for a regulator of the GlnA protein in response to the levels of nitrogen.

**Figure 5:**
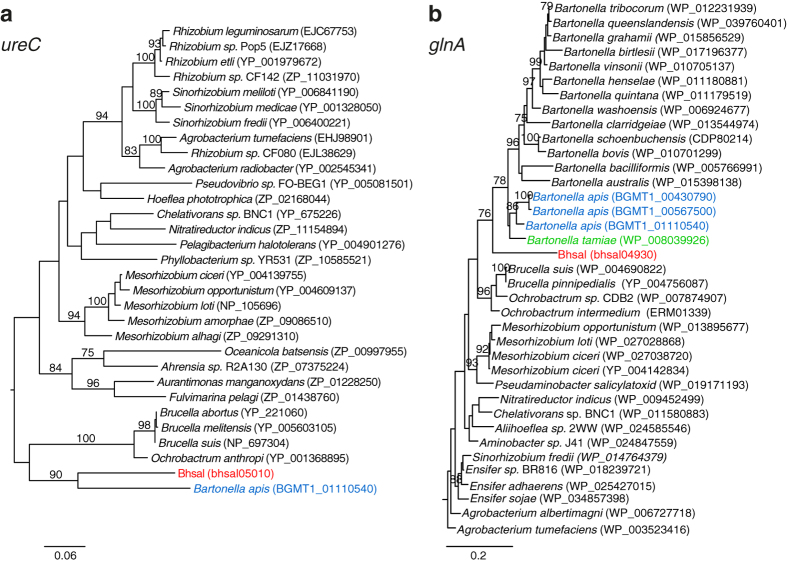
Phylogenetic inference of urease and glutamine synthetase. Phylogenies of (**a**) the urease subunit alpha (*ureC*) and (**b**) and glutamine synthetase (*glnA*), based on their protein sequences. Accession numbers for each sequence are shown in parentheses. Only bootstrap values higher than 75% are shown. Red color represents the Bhsal sequences; green, *B. tamiae*; and blue, *B. apis*. The outgroup sequences were removed to aid visualization, these being: (**a**) AEV60653, CAH36667 and AF411018 from *Pseudomonas fluorescens, Burkholderia pseudomallei* and *Nitrosospira sp*. NpAVin, respectively, and (**b**) YP_008745196 and EAP72618 from *Burkholderia pseudomallei* and *Ralstonia solanacearum,* respectively. The figure was drawn with Figtree (Andrew Rambaut, available on the author’s website: http://tree.bio.ed.ac.uk/software/figtree/), and edited with Adobe Illustrator.

The urease genes were also identified in *B. apis*, another gut bacterium, as inferred from an analysis of sequences from the metagenome of the Alpha-1 bin of the *A. mellifera* gut microbiota. Furthermore, we identified the urease genes in *Brucella*, which has a fecal-oral transmission pathway in mammals. Single protein phylogenies of the *ureC* and *glnA* gene products confirmed that Bhsal and *B. apis* clusters with *Brucella* (Fig. 5), which suggests that the identified urease genes in these species share a common ancestry and have been vertically inherited.

It has been shown that *Blattabacterium* strain Bge, which is the primary endosymbiont of the cockroach *Blatella germanica*, contains the urease genes but not the gene for the glutamine synthetase^31^. In effect, ammonia and carbon dioxide are the final catabolic products of amino acids in this species. Interestingly, it has been shown that the ammonium produced during degradation of urea protects *Brucella* species against the acidic conditions in the animal stomach^32^. If glutamine is provided by the ant diet, the *glnA* gene may be downregulated resulting in increased levels of ammonia, especially since the *carAB* genes have been lost which prevents conversion of ammonia to carbamoyl-phosphate. Depending on species the pH of the ant gut has been shown to range from slightly acidic or neutral pH^33^, to as low as pH 3 in the rectum of leafcutter ants^34^ and thus the production of ammonium may raise the pH locally, contributing to a microenvironment where Bhsal can cope.

In contrast, the urease genes could not be identified in the canonical *Bartonella* species, or in *B. tamiae*. These bacterial species do not pass through the stomach of the mammalian host, but are instead transmitted to novel hosts via blood-sucking insects. It has been shown that an inactivating mutation in the *ureD* gene has facilitated blood-borne transmission pathways of *Yersinia pestis* in fleas^35^,^36^. About 30–40% of the fleas infected with *Yersinia pseudotuberculosis*, which contains the urease function, show signs of disease, including diarrhea, immobility and death after a blood meal. These disease symptoms are however not observed in *Y. pestis*-infected fleas in which the urease function has been inactivated. By analogy, inactivation and loss of the urease function may have facilitated the adaptation of *Bartonella* species to blood-sucking insects.

Thus, we hypothesize that the loss of the urease gene cluster facilitated blood-borne transmission pathways and thereby the spread of *Bartonella* to a broad diversity of mammalian hosts. Our phylogenetic inference suggests that *B. tamiae* is a sister species to *B. apis* rather than to the canonical *Bartonella* species, which indicates convergent losses of the urease genes and independent adaptations to blood-borne transmission pathways in these lineages. The presence of the urease functions in *Brucella* has likely enabled oral transmission pathways^32^, whereas its absence from *Bartonella* may have facilitated blood-borne transmission pathways. In summary, we suggest that the presence versus the absence of the urease function has played an important role for lifestyle switches in the host-associated members of the Rhizobiales group of bacteria.

### Species designation

The level of sequence divergence in the 16 S rRNA genes of Bhsal and its closest relatives, the *Bartonella spp.*, ranges from 4.5% to 5.9%. This is well above the sequence divergence level normally used for species designations, and also above the common 5% divergence for a genus^37^. Thus, we suggest that Bhsal represents a new species of a distinct genus, and should be named accordingly. We propose that Bhsal should be given the name ‘*Candidatus* Tokpelaia hoelldoblerii strain Hsal’. The genus name is a latinized version of Tokpela, the First World of Hopi cosmogony. During Tokpela’s destruction by fire the Ant People sheltered the First People. The proposed genus name refers to the resemblance of the hosting provided by ants to the bacterium. The species name is taken from the German myrmecologist Bert Hölldobler who contributed extensively to the knowledge of ant biology.

## Methods

### Gap closure and genome assembly

Detailed information on the sequencing and initial assembly of the draft genome can be found in ref. 11. Briefly, the host genome was sequenced with both short and long paired-end reads (insert sizes ranging from 200 bp to 10 kb), which allowed to obtain a single, circular scaffold consisting of 119 contigs, unambiguously placed and oriented. To close the contig gaps of the Bhsal draft genome, whole *H. saltator* ants were collected from the colonies that were used in the sequencing project. Whole ant abdomens were crushed in liquid nitrogen and Phenol-chloroform/IAA extraction was used to extract the total DNA for the gap closure PCR reactions and stored in −20 °C. The 1.86 Mb bacterial scaffold had 119 gaps, and primer pairs were designed for each gap using sequences located 200 bp from the contig ends as templates. Nine additional primer pairs were designed in a second step for PCR reactions that did not yield a product or gave unspecific products when visualized on the agarose gel.

All PCR products were sequenced with the Sanger method, assembled and trimmed for low quality bases and added to the draft genome using Phred, Phrap^38^,^39^ and Consed^40^. An in-house perl script was used to check the quality of the resulting assembly and fifteen low-quality areas were detected with too low, too high or no coverage and inconsistent read-pairs (Supplementary Table S8). For these ambiguous areas, new PCR primers were designed and the intervening segments were amplified by PCR, sequenced and trimmed as described above. Four regions remained unresolved and the PCR products over these regions were nebulized, end-repaired, purified and cloned into the pSMART-HCkan vector (Lucigen) and sequenced by the Sanger sequencing method. The obtained sequences were assembled with the draft genome using Phred, Phrap and Consed. Finally all sequences were visually aligned to the Bhsal genome in Artemis^41^, ensuring that the sequence spanned the original gap or ambiguous area before merging it with the draft genome.

All 25 μl PCR reactions contained 0.8 μM of each primer, 0.4 mM dNTP mix, 1x AccuTaq™ buffer, 0.05 U/μl of AccuTaq LA DNA polymerase, approximately 0.1 ng/μl of template DNA and H_2_O was added to reach the final volume of 25 μl. Reagents, enzyme and primers were from Sigma. The PCR protocol was as follows: initial denaturation 30 s in 96 °C, followed by 33 cycles at 94 °C for 30 s, 55 °C for 45 s, 68 °C for 2 min. Final extension was in 68 °C for 10 min. The sizes of the PCR products were verified on a 1% agarose gel in 1x TAE buffer and further purified for sequencing either with QIAquick^®^ PCR Purification kit (QIAGEN) or Illustra™ GFX™ PCR DNA and Gel Band Purification Kit (GE healthcare). Samples were stored in −20 °C.

The sequencing was carried out with using BigDye^®^ Terminator v3.1. Cycle Sequencing kit (Applied Biosystems). Each sequencing reaction composed of 0.5 μl BigDye Ready Reaction Mix, 1.75 μl BigDye Sequencing Buffer, ~20 ng of template DNA, 0.25 μM of the corresponding PCR primer and H_2_O was added to reach the volume of 10 μl. Thermal cycling protocol was as follows: 95 °C for 30 s, 30 cycles in 94 °C for 25 s, in 50 °C for 15 s and 60 °C for 2 min. The sequencing products were purified with Sephadex™ G-50 (GE Healthcare) and Sanger sequenced with ABI 3730xl (Applied Biosystems). All samples were stored in −20 °C.

### Genome annotation

The origin and terminus of replication were determined by calculating the cumulative GC skew. The exact location of the terminus was determined by the identification of the *dif*-site. To this end, a consensus *dif* sequence was constructed from an alpha-proteobacterial dataset^13^ using Ambiguity Consensus Maker (http://www.hiv.lanl.gov/content/sequence/CONSENSUS/AmbigCon.html) and used as a query for the identification of it’s location in the Bhsal genome. The Bhsal genome was annotated using the manually annotated *B. grahamii* core genome as a reference, as described previously^9^. Blast searches against the COG database was performed for all CDSs, and a COG was assigned to a CDS whenever the two best Blast hits belonged to the same COG (E < 0.01). GC3s values for the CDSs were calculated using the codonw package^42^. BlastKOALA was used to predict genes involved in amino acid and vitamin biosynthetic pathways^43^. The *cas* genes associated with the two CRISPR systems in Bhsal were detected in the annotation process and to specify the spacers we used CRISPRFinder available online^26^. The spacers from both systems were blasted against the NCBIs nonredundant nucleotide (nr) database and a local database consisting of the *Bartonella* genomes (Supplementary Table S2) using blastn with default parameters.

### Protein family clustering and phylogenetic analyses

For the 16 S rRNA phylogeny includ, we extracted sequences from eight canonical *Bartonella* sequences plus *B. tamiae*. Related 16 S rRNA sequences from arthropods were retrieved from NCBI using the accession numbers taken from refs 6 and 7. The selected sequences were aligned with Mafft-linsi and the columns that had more than 50% gaps were trimmed with trimAl^44^. A phylogeny was inferred with RAxML using the GTRGAMMA model with 100 bootstrap replicates.

To extract protein sequences for the clustering, all-against-all blastp^45^ searches were performed with all proteins encoded by the Bhsal genome, ten canonical *Bartonella* genomes, two *B. tamiae* genomes (strains Th239 and Th307), one metagenome from *B. apis*, and the genomes of six outgroup species with an E-value cutoff of 10^−5^ (Supplementary Table S2). The extracted proteins were clustered into families using orthoMCL^46^. In total, 629 protein families contained a single protein from each taxa and at least one protein from the *B. apis* metagenome. For each family, the identified proteins were aligned with Mafft-linsi^47^, and trimmed off for all sites with more than 50% of gaps using trimAl^44^. Phylogenetic trees were inferred based on all proteins in each family using the PROTCATLG model in RAxML^48^ by generating 100 rapid bootstrap trees and one slower and more thorough search. The trees were manually examined for monophyly of all *B. apis* metagenome sequences. Only 8 trees indicated paraphyly of this group with the inclusion of *B. tamiae* (>75% bootstrap support). The longest *B. apis* metagenome sequence in the clade was selected to represent *B. apis*. Finally, a concatenated alignment of the 629 clusters was constructed using custom perl scripts, from which a tree was inferred using the RAxML strategy described above^48^.

A second orthoMCL run was done with the exclusion of the proteins encoded by the *B. apis* metagenome and one of the *B. tamiae* proteomes. In this analysis, 630 single-copy panortholog clusters were identified. *B. bacilliformis* contained a recent duplication of *rplB, rplC, rplD, rplN, rplP, rplV, rplW, rplX, rpmC, rpsC, rpsG, rpsJ, rpsQ* and *rprS*. Additionally, *groEL* and *groES* contained several paralogs in the outgroup taxa. These 17 protein clusters were aligned, trimmed, and used for phylogenetic inference. These trees confirmed that the duplicated copies clustered and the gene copy associated with the shortest branch in the tree was retained, resulting in a dataset of 647 protein families. The proteins in these families were aligned with Mafft-linsi^47^, trimmed with BMGE^49^ with default parameters, and used for phylogenetic inference with RAxML^48^, both individually and after concatenation. The 647 untrimmed protein alignments were converted back to their nucleotide sequences and each protein family was categorized based on the GC bias at the first and second codon positions, defined as


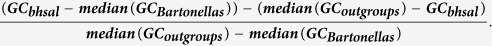


The phylogeny of urease (UreC) and glutamine synthase (GlnA), were produced from protein sequences obtained by blastp^45^ against NCBI’s nr database with default parameters. The significant hits were aligned using Mafft-linsi, trimmed with trimAL for sites with over 50% gaps and phylogenetic trees were constructed using RAxML PROTGAMMALG with 100 bootstraps.

### Gene flux analyses

The protein families produced by OrthoMCL^46^ were mapped onto the phylogeny using generalized parsimony with ACCTRAN in PAUP* 4.0b10^50^ with the following penalties: 10 for an ortholog gain, 5 for ortholog loss, 1 for gene duplication and 0.2 for other copy-number variation. These changes in the orthologous groups were mapped onto the concatenated core gene tree of the 17 genomes.

### Data accessibility

The genome sequence data has been deposited at the European Bioinformatics Infrastructure (EBI) and the National Center for Bioinformatics Infrastructure (NCBI) and assigned the accession number CP017315.

## Additional Information

**How to cite this article**: Neuvonen, M.-M. *et al*. The genome of Rhizobiales bacteria in predatory ants reveals urease gene functions but no genes for nitrogen fixation. *Sci. Rep.* **6**, 39197; doi: 10.1038/srep39197 (2016).

**Publisher's note:** Springer Nature remains neutral with regard to jurisdictional claims in published maps and institutional affiliations.

## Supplementary Material

Supplementary Information
