# Supplementary material for: The genome of Rhizobiales bacteria in predatory ants reveals urease gene functions but no genes for nitrogen fixation
Source: Sci Rep. 2016 Dec 15;6:39197. doi: 10.1038/srep39197 (PMC5156944; doi:10.1038/srep39197)
Supplement: Supplementary Information [file srep39197-s1.pdf]

Supplementary information

The Genome of Rhizobiales Bacteria in Predatory Ants Reveals Urease Gene Functions but no Genes for Nitrogen Fixation

Minna-Maria Neuvonen, Daniel Tamarit, Kristina Näslund, Juergen Liebig, Heike Feldhaar, Nancy Moran, Lionel Guy, Siv G. E. Andersson

Supporting information

Fig. S1 Phylogeny based on the 16S rRNA gene. Phylogeny based on the 16S rRNA gene inferred with the maximum likelihood method. Only bootstrap values above 75% are shown. Sequence names composed of the host species name, instead of the bacterial species name, are underlined. The tribe and the subfamily of the hosts are shown, highlighted in yellow if they belong to the Apini, in red if they belong to the Formicoid clade, and in orange if they belong anywhere else in the crown ants group. The tree was drawn using Figtree (Andrew Rambaut, available on the author's website: http://tree.bio.ed.ac.uk/software/figtree/), and clade colors were added using Adobe Illustrator.

Fig. S2 Phylogenetic and gene flux analyses. (a) The reference phylogeny of Bhsal, *B. tamiae* and the canonical *Bartonella* species was inferred from a concatenated protein data set. Bhsal is highlighted in red. Dashed squares above key branches represent the percentage of single gene trees that include those branches with high bootstrap support (>70%), out of the total of 647 single copy panorthologs. The tree was drawn using Figtree (Andrew Rambaut, available on the author's website: http://tree.bio.ed.ac.uk/software/figtree/) and the squares were added using Adobe Illustrator. (b) Gains and losses of protein families were mapped onto the reference phylogeny shown in (a). Above each branch, gains of protein families are shown in green, while losses are shown in red. Beneath each branch, the total number of protein families is shown. The tree and coloring was drawn using custom perl and bash scripts.

-

Fig. S3 CRISPR cas regions. Figure drawn to scale using GenoplotR (Guy et al. 2010)

Table S1 Sequence similarity of 16S rRNA genes .The table shows the percent sequence identity (% id) and the length of alignments in pairwise comparisons of the 16S rRNA genes in Bhsal and other bacterial species.

Table S2 Genome data. Accession numbers, genome sizes, GC contents and abbreviations are shown for the genomes selected for the analyses reported in this manuscript.

Table S3. Least biased genes. List of the 50 least GC12-biased genes in Bhsal and the associated phylogenetic support for the specified topologies, with a bootstrap support higher than 70%.

Table S4 List of ancestrally acquired genes. The list includes genes inferred as gained in the common ancestor of Bhsal, *Bartonella* and *B. tamiae*.

Table S5 List of genes acquired in the ancestor of *Bartonella*.

Table S6 List of genes acquired in the ancestor of Bhsal.

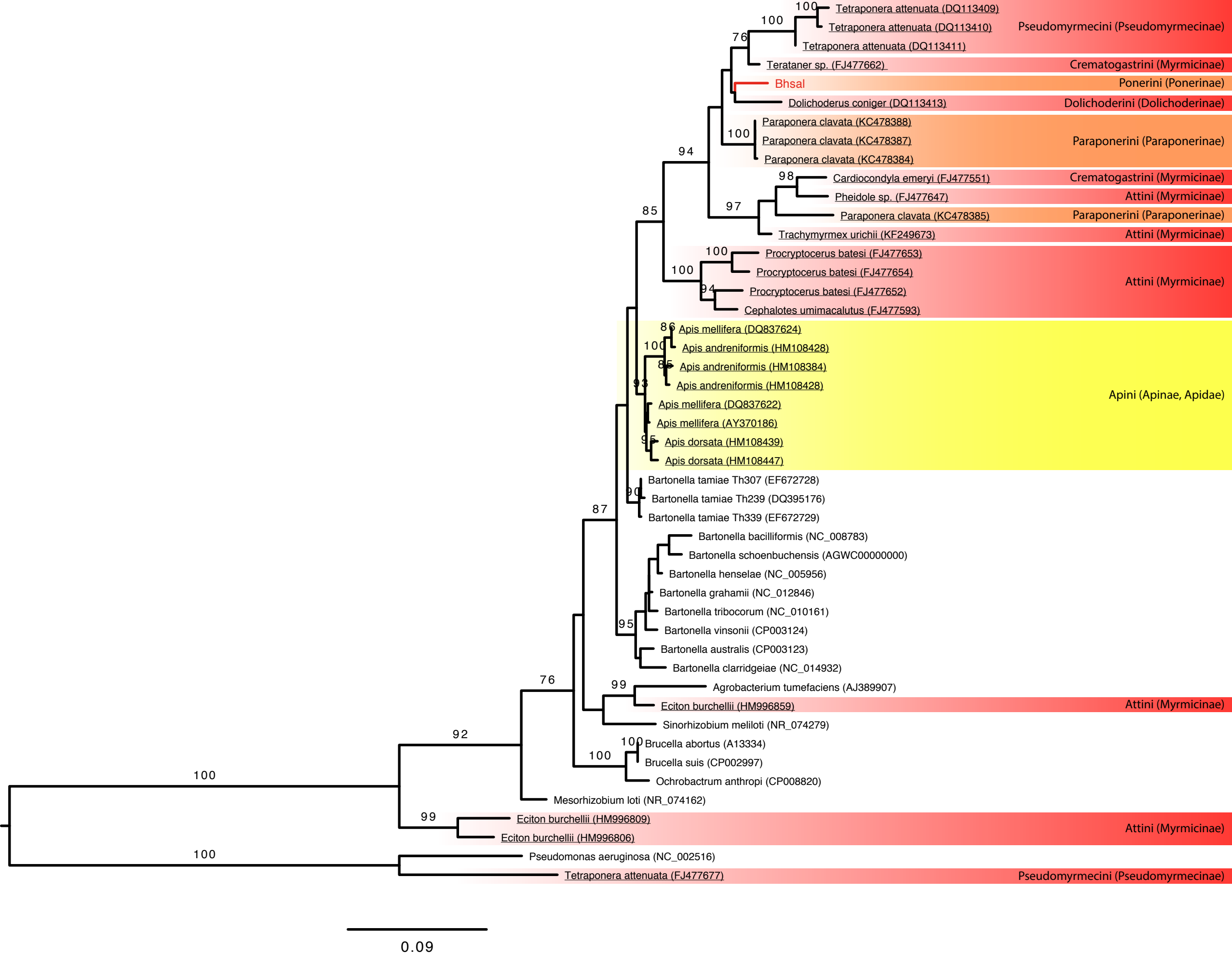

Tetraponera attenuata (DQ113409)
Tetraponera attenuata (DQ113410)
Tetraponera attenuata (DQ113411)
Pseudomyrmecini (Pseudomyrmecinae)
Arboreal omnivores
Terataner sp. (FJ477662)
Crematogastrini (Myrmicinae)
Bhsal
Ponerini (Ponerinae)
Dolichoderus coniger (DQ113413)
Dolichoderini (Dolichoderinae)
Paraponera clavata (KC478388)
Paraponera clavata (KC478387)
Paraponera clavata (KC478384)
Paraponerini (Paraponerinae)
Cardiocondyla emeryi (FJ477551)
Crematogastrini (Myrmicinae)
Pheidole sp. (FJ477647)
Attini (Myrmicinae)
Paraponera clavata (KC478385)
Paraponerini (Paraponerinae)
Trachymyrmex urichii (KF249673)
Attini (Myrmicinae)
Procryptocerus batesi (FJ477653)
Procryptocerus batesi (FJ477654)
Procryptocerus batesi (FJ477652)
Cephalotes umimacalutus (FJ477593)
Attini (Myrmicinae)
Apis mellifera (DQ837624)
Apis andreniformis (HM108428)
Apis andreniformis (HM108384)
Apis andreniformis (HM108428)
Apis mellifera (DQ837622)
Apis mellifera (AY370186)
Apis dorsata (HM108439)
Apis dorsata (HM108447)
Apini (Apinae, Apidae)
Bartonella tamiae Th307 (EF672728)
Bartonella tamiae Th239 (DQ395176)
Bartonella tamiae Th339 (EF672729)
Bartonella bacilliformis (NC_008783)
Bartonella schoenbuchensis (AGWC00000000)
Bartonella henselae (NC_005956)
Bartonella grahamii (NC_012846)
Bartonella tribocorum (NC_010161)
Bartonella vinsonii (CP003124)
Bartonella australis (CP003123)
Bartonella clarridgeiae (NC_014932)
Agrobacterium tumefaciens (AJ389907)
Eciton burchellii (HM996859)
Attini (Myrmicinae)
Sinorhizobium meliloti (NR_074279)
Brucella abortus (A13334)
Brucella suis (CP002997)
Ochrobactrum anthropi (CP008820)
Mesorhizobium loti (NR_074162)
Eciton burchellii (HM996809)
Eciton burchellii (HM996806)
Attini (Myrmicinae)
Pseudomonas aeruginosa (NC_002516)
Tetraponera attenuata (FJ477677)
Pseudomyrmecini (Pseudomyrmecinae)
0.09

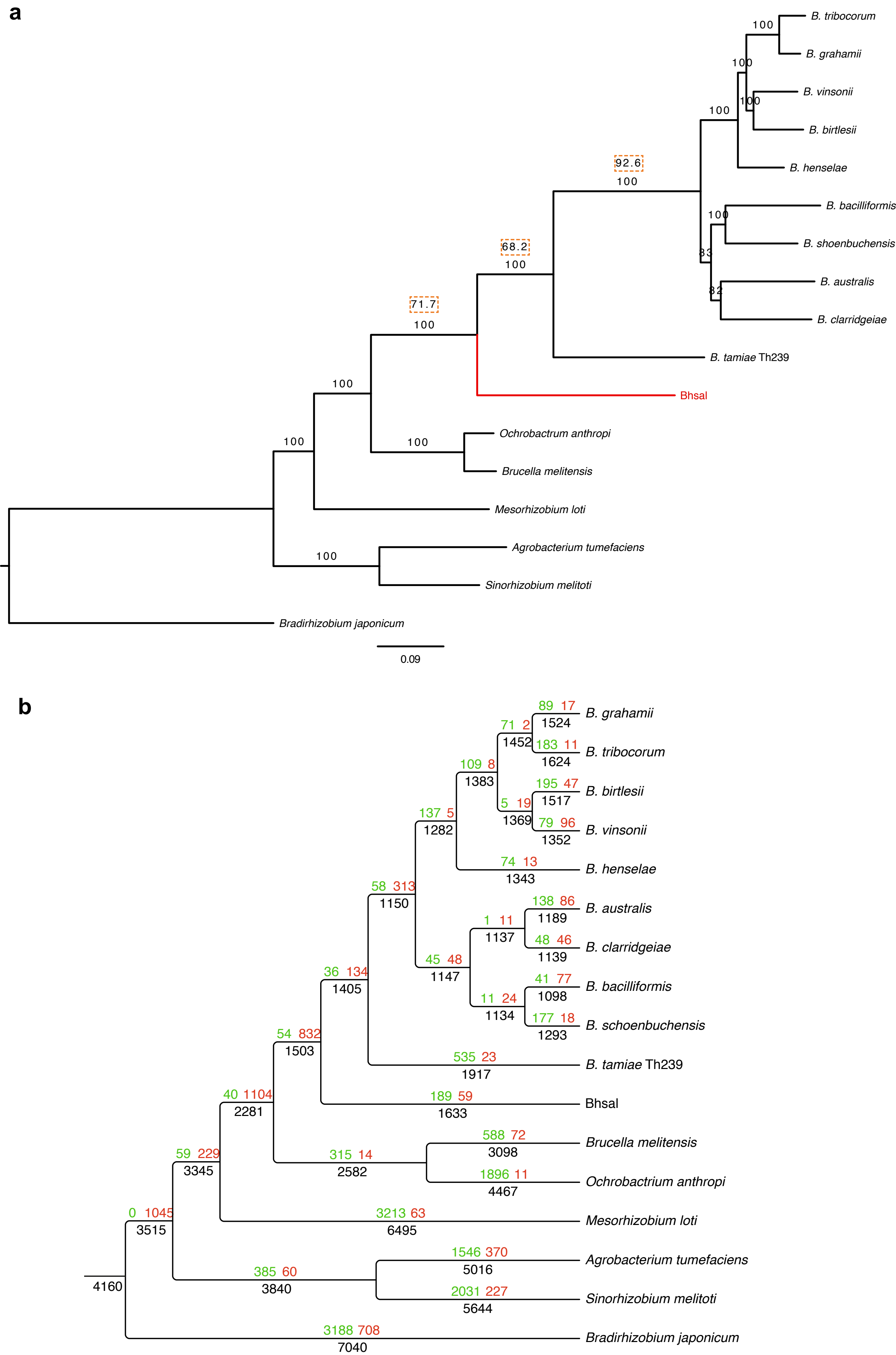
a
B. tribocorum
B. grahamii
B. vinsonii
B. birtlesii
B. henselae
B. bacilliformis
B. shoenbuchensis
B. australis
B. clarridgeiae
B. tamiae Th239
Bhsal
Ochrobactrum anthropi
Brucella melitensis
Mesorhizobium loti
Agrobacterium tumefaciens
Sinorhizobium melitoti
Bradirhizobium japonicum
92.6
68.2
71.7
0.09
b
B. grahamii
B. tribocorum
B. birtlesii
B. vinsonii
B. henselae
B. australis
B. clarridgeiae
B. bacilliformis
B. schoenbuchensis
B. tamiae Th239
Bhsal
Brucella melitensis
Ochrobactrum anthropi
Mesorhizobium loti
Agrobacterium tumefaciens
Sinorhizobium melitoti
Bradirhizobium japonicum

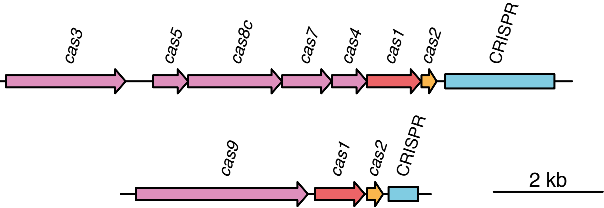

cas3
cas5
cas8c
cas7
cas4
cas1
cas2
CRISPR
cas9
cas1
cas2
CRISPR
2 kb

Supplementary Table S1. Sequence similarity of 16S rRNA genes against Bhsal.

Subject id	% id	Alignment length	Mis-matches	Gaps	Query start	Query end	Subject start	Subject end	E-value	Bit score
Ärataner sp. FJ477662 ★	97,49	1354	33	1	48	1400	1	1354	0	2311
Dolichoderus coniger DQ113413 ★	96,97	1321	39	1	70	1390	1	1320	0	2217
Paraponera clavata PT95A1a KC478387 ★	96,78	1057	33	1	73	1128	1	1057	0	1762
Paraponera clavata T51A1a KC478388 ★	96,77	1053	33	1	73	1124	1	1053	0	1755
Paraponera clavata 13Ali KC478384 ★	96,62	1036	34	1	73	1108	1	1035	0	1718
Trachymyrmex urichii KF249673 ★	95,80	1451	56	3	2	1448	1	1450	0	2337
Tetraponera attenuata DQ113411 ★	95,76	1321	51	5	70	1389	1	1317	0	2124
Tetraponera attenuata DQ113409 ★	95,63	1235	51	3	84	1316	1	1234	0	1982
Bartonella tamiae Th339 EF672729	95,46	1211	53	2	76	1284	1	1211	0	1930
Bartonella tamiae Th307 EF672728	95,40	1477	61	3	1	1472	1	1475	0	2344
Tetraponera attenuata DQ113410 ★	95,39	1322	57	4	70	1389	1	1320	0	2108
Bartonella tamiae Th239 DQ395176	95,34	1222	55	2	73	1292	4	1225	0	1940
Apis mellifera AY370186 ★	95,32	1454	61	4	1	1449	2	1453	0	2302
Pheidole sp. FJ477647 ★	95,28	1357	61	3	73	1426	69	1425	0	2148
Bartonella tribocorum	95,06	1478	67	5	1	1472	1	1478	0	2320
Bartonella grahamii	94,99	1477	69	5	1	1472	1	1477	0	2313
Apis mellifera DQ837622 ★	94,91	1297	59	4	21	1312	1	1295	0	2023
Bartonella bacilliformis	94,79	1421	70	4	1	1417	1	1421	0	2211
Bartonella henselae Houston1	94,72	1476	74	4	1	1472	1	1476	0	2290
Bartonella australis NH1	94,71	1474	75	3	1	1472	1	1473	0	2287
Apis dorsata HM108439 ★	94,68	1446	70	4	2	1442	1	1444	0	2237
Apis andreniformis HM108428 ★	94,61	1446	71	4	2	1442	2	1445	0	2231
Apis andreniformis HM108384 ★	94,59	1442	71	4	6	1442	9	1448	0	2224
Mesorhizobium loti MAFF303099 NR 074162	94,59	1460	78	1	14	1472	1	1460	0	2257
Bartonella vinsonii berkhoffii winnie	94,56	1488	65	10	1	1472	1	1488	0	2285
Bartonella clarridgeiae	94,51	1476	77	4	1	1472	1	1476	0	2274
Cardiocondyla emeryi FJ477551 ★	94,50	1401	69	5	21	1414	1	1400	0	2154
Apis dorsata HM108447 ★	94,50	1381	68	5	1	1375	6	1384	0	2122
Bartonella schoenbuchensis m07a	94,45	1477	77	5	1	1472	1	1477	0	2268
Apis mellifera DQ837624 ★	94,30	1297	67	4	21	1312	1	1295	0	1978
Apis mellifera DQ837623 ★	94,14	1297	69	4	21	1312	1	1295	0	1967
Sinorhizobium meliloti 1021 NR 074279	94,04	1476	81	6	1	1472	8	1480	0	2231
Brucella suis 1330 CP002997	93,97	1477	80	5	1	1472	9	1481	0	2226
Brucella abortus A13334 CP003176	93,97	1477	80	5	1	1472	1	1473	0	2226
Procryptocerus batesi FJ477652 ★	93,97	1426	66	7	21	1426	1	1426	0	2139
Paraponera clavata 15Ali KC478385 ★	93,83	1037	62	2	73	1108	1	1036	0	1559
Cephalotes unimacalutus FJ477593 ★	93,70	1428	66	12	21	1426	1	1426	0	2117
Eciton burchellii HM996859 ★	93,61	1409	85	4	21	1426	1	1407	0	2098
Procryptocerus batesi FJ477653 ★	93,32	1423	75	11	24	1426	4	1426	0	2084
Ochrobactrum anthropi R0018 CP008820	93,22	1474	95	5	1	1472	1	1471	0	2163
Procryptocerus batesi FJ477654 ★	92,93	1429	76	16	21	1426	1	1427	0	2056
Agrobacterium tumefaciens 0362 AJ389907	92,63	1438	103	3	21	1456	1	1437	0	2065
Eciton burchellii HM996806 ★	90,22	1411	126	11	21	1426	1	1404	0	1831

Eciton burchellii HM996809 ★	90,02	1413	125	11	21	1426	1	1404	0	1814
Pseudomonas aeruginosa PAO1 NC 002516	84,14	1059	141	22	427	1472	487	1531	0	1000
Tetraponera attenuata FJ477677 ★	82,96	1250	159	30	183	1393	197	1431	0	1086

★: unclassified bacterial species. The species designation belongs to the arthropod host whose metagenome yielded the specified hit.

Supplementary Table S2. Genome data

Organism	Lifestyle*	Host	GC%	Chromosome Size (Mb)	Accession number	Reference
Candidatus Tokpelaia hoelldoblerii Hsal	M?	Hymenoptera (Harpegnathos)	54	1,9	CP017315	**This study**
Bartonella apis	M	Hymenoptera (Apis)	44	NA	2166559100**	Kesnerova et.al. (2016). Int J System Evo. Microbio.**66**, 414-421
Agrobacterium tumefaciens C58	P	Various plants	59	2.8	NC_003062	Wood et al. (2001), Science, **294**:2317-23: Goodner et al (2001) Science, **294:** 2323-8
Sinorhizobium meliloti 1021	M	Legumes	62	3,7	NC_003047	Capela et al. (2001) PNAS, 98: 9877-82; Galibert et al (2001) Science, **293:** 668-72
Mesorhizobium loti MAFF303099	M	Legumes	62	7	NC_002678	Kaneko et al. (2000). DNA Res., **7**: 331-8
Brucella melitensis 16M	H	Sheep, cattle	57	chr1 2.1<br>chr2 1.2	NC_003317<br>NC_003318	DelVecchio et al. (2002) PNAS, **99:** 443-8
Ochrobactrum anthropi ATCC 49188	H	Various	56	chr1 2.9<br>chr2 1.9	NC_009667<br>NC_009668	Chain et al. 2011, J Bacteriol., **193:** 4274-5
Bradyrhizobium japonicum USDA 110	M	Legumes	64	9,1	NC_004463	Kaneko et al. (2002) DNA Res., **9:** 189-97
B. australis		Kangaroo (*Macropus giganteus*)	42	1,6	CP003123	Fournier et al. (2007) Emerg Infect Dis, **13:** 1961-1962.
B. bacilliformis KC583	H	Human (*Homo sapiens*)	38	1,5	NC_008783	Carrasco SE, et al. (2014) Vet Microbiol, **170:** 325-334.
B. schoenbuchensis m07a		Moose (*Alces alces*)	38	1,7	AGWC00000000	Guy et al. (2013) *PLoS Genet*, **9**: e1003393
B. clarridgeiae 73		Cat (*Felis catus*)	36	1,5	NC_014932	Engel et al. (2011) PLoS Genet, **7:** e1001296
B. henselae Houston-1	H	Human (*Homo sapiens*)***	38	1,9	NC_005956	Alsmark et al. (2004) *Proc Natl Acad Sci U S A*, **101**: 9716-9721.
B. vinsonii berkhoffii Winnie		Dog [Pekingese] (*Canis lupus*)	39	1,8	CP003124	Kordick and Breitschwerdt (1998) *Emerg Infect Dis*, **4**: 325-328.
B. grahamii as4aup		Wood mouse (*Apodemus sylvaticus*)	38	2,4	NC_012846	Berglund et al. (2009) *PLoS Genet*, **5**: e1000546.
B. tribocorum IBS 325		Rat (*Rattus norvegicus*)	39	2,6	NC_010161	Saenz et al. (2007) *Nat Genet*, **39**: 1469-1476.
B. birtlesii IBS325	H	Rodents (Apodemus)	37	1,8	NZ_AKIP00000000	Rolain et al. (2012) J Bacteriol, **194:** 4779
B. tamiae Th239	H	?	39	2,2	NZ_AIMB00000000	NA
B. tamiae Th307	H	?	39	2,2	NZ_AIMG00000000	NA

(*) Lifestyle types: (P) Plant pathogen; (M) Mutualistic; (H) Human pathogen.

(**) The alpha-1 metagenome BIN ID from IMG/JGI

(***) Cats are the supposed natural reservoir of *B. henselae*

Supplementary table S3. Least biased genes. List of the 50 least GC12-biased genes in bhsal and the associated phylogenetic support for the specified topologies, with a bootstrap support higher than 70%.

Locus tag	Gene name	Product	Length (bp)	location bhsal	location BG	location BAnh1	GC12 Bhsal	GC12 Bartonellas	GC12 Outgroups	Barto[a]	(Barto, Bt)[b]	((Barto,Bt), bhsal)[c]
bhsal05240	*rpsJ*	30S ribosomal protein S10	306	560893	1484749	983116	0,4854	0.4898+-0.0048	0.5016+-0.0122			
bhsal06890		Uncharacterized protein	366	726058	1315808	719177	0,4797	0.4806+-0.0152	0.4907+-0.0146			
bhsal07630	*ihfA*	Integration host factor subunit alpha	309	813580	1283159	749199	0,4615	0.4708+-0.0131	0.4983+-0.0126	✓		
bhsal13340		Uncharacterized protein	426	1457439	601370	469618	0,4441	0.4527+-0.0068	0.5235+-0.0223	✓	✓	
bhsal01330		Uncharacterized protein	471	140704	2137596	1444454	0,4494	0.4531+-0.0251	0.5433+-0.0404	✓		
bhsal05290	*rpsS*	30S ribosomal protein S19	276	563753	1481892	980255	0,4785	0.4769+-0.0067	0.4865+-0.0176			
bhsal00190	*ihfB*	Integration host factor subunit beta	285	20654	12736	11617	0,4948	0.4943+-0.0134	0.5299+-0.0212	✓		
bhsal11670		Hypothetical cytosolic protein	498	1263028	660264	1118054	0,4611	0.4545+-0.0113	0.5138+-0.0184	✓	✓	✓
bhsal00630	*rpmI*	50S ribosomal protein L35	198	61396	127150	98074	0,4328	0.4279+-0.0108	0.4617+-0.0119			
bhsal02650		Glyceraldehyde-3-phosphate dehydrogenase	1005	262065	2131067	1439343	0,4985	0.4942+-0.0065	0.5126+-0.0076	✓		
bhsal10080		Glutathione S-transferase	690	1086306	1074460	1181058	0,513	0.4993+-0.0089	0.5621+-0.0091	✓	✓	
bhsal07720	*ppnK*	Probable inorganic polyphosphate-ATP-NAD kinase	762	825837	888655	715067	0,4902	0.4765+-0.0162	0.5482+-0.0361	✓		
bhsal11390	*rpsT*	30S ribosomal protein S20	264	1231359	152899	137244	0,5281	0.5107+-0.0210	0.5693+-0.0235	✓		
bhsal03270	*atpG*	ATP synthase gamma chain	903	339160	2165900	1462964	0,495	0.4850+-0.0090	0.5173+-0.0124	✓		
bhsal02870		Glutaredoxin- GrxC family	252	290303	526165	402469	0,4588	0.4436+-0.0247	0.5228+-0.0181	✓	✓	
bhsal00280	*rplS*	50S ribosomal protein L19	447	26466	2234929	1507556	0,5433	0.5261+-0.0160	0.5726+-0.0322			
bhsal13880	*rpsU*	30S ribosomal protein S21	213	1514602	171994	259773	0,5486	0.5306+-0.0153	0.5815+-0.0416			
bhsal00270	*trmD*	tRNA (guanine-N(1)-)-methyltransferase	696	25571	2235838	1508470	0,5515	0.5296+-0.0126	0.5969+-0.0289	✓	✓	✓
bhsal05380	*rpsN*	30S ribosomal protein S14	303	567509	1478183	976543	0,5098	0.5029+-0.0106	0.5237+-0.0114			
bhsal07640		Regulatory protein MerR	522	813937	1282650	749712	0,4686	0.4321+-0.0202	0.5396+-0.0173	✓	✓	
bhsal01890		Uncharacterized protein	528	195114	2107697	1428071	0,5056	0.4664+-0.0207	0.5512+-0.0195	✓		
bhsal14710	*infA*	Translation initiation factor IF-1	216	1608596	1983324	1384831	0,4315	0.4062+-0.0092	0.4842+-0.0414			
bhsal05300	*rplV*	50S ribosomal protein L22	387	564033	1481611	979974	0,5154	0.4981+-0.0077	0.5372+-0.0153			
bhsal12140		Folate-binding protein YgfZ	879	1328819	725955	554877	0,4779	0.4076+-0.0104	0.5739+-0.0240	✓	✓	✓
bhsal08690		CDP-diacylglycerol--glycerol-3-phosphate 3-phosphatidyltransferase	594	933103	774132	605694	0,4673	0.4491+-0.0094	0.5002+-0.0104	✓	✓	
bhsal03290	*atpC*	ATP synthase epsilon chain	426	341648	2163235	1460391	0,5035	0.4809+-0.0114	0.5392+-0.0217	✓		
bhsal02960		Uncharacterized protein	1950	301771	1899954	1354369	0,4923	0.4542+-0.0108	0.5459+-0.0133	✓	✓	✓

bhsal03360	*pncB*	Nicotinate phosphoribosyltransferase	1302	350005	115875	108528	0,4805	0.4572+-0.0097	0.5111+-0.0073	✓	✓	
bhsal16140	*purE*	N5-carboxyaminoimidazole ribonucleotide mutase	495	1764086	1989816	1385187	0,5633	0.5324+-0.0177	0.6028+-0.0122	✓		
bhsal13850		invasion associated locus B family protein	573	1511371	572556	444134	0,5026	0.4509+-0.0169	0.5725+-0.0179			
bhsal11190		Uncharacterized protein	924	1211613	1082215	1175430	0,5324	0.4723+-0.0099	0.6044+-0.0129	✓	✓	
bhsal08130		Cyclase-dehydrase	408	875097	804246	632913	0,4343	0.3925+-0.0183	0.4841+-0.0223	✓	✓	✓
bhsal07360	*cdsA*	Phosphatidate cytidylyltransferase	855	784220	858647	686518	0,4773	0.4098+-0.0157	0.5724+-0.0254	✓	✓	
bhsal08880		Single-stranded-DNA-specific exonuclease RecJ	1803	950944	1428827	879789	0,539	0.4886+-0.0154	0.5932+-0.0121	✓	✓	✓
bhsal11310		Uncharacterized protein	609	1222107	161601	146022	0,4534	0.4137+-0.0072	0.4952+-0.0080	✓	✓	✓
bhsal16110		ATP-dependent protease	681	1763468	1774620	1264351	0,4846	0.4310+-0.0142	0.5447+-0.0163	✓	✓	✓
bhsal03280	*atpD*	ATP synthase subunit beta	1494	340083	2164969	1462033	0,5251	0.5047+-0.0080	0.5471+-0.0079	✓		
bhsal08540		Uncharacterized protein	1182	918166	757813	586334	0,462	0.4120+-0.0189	0.5112+-0.0122	✓	✓	✓
bhsal06170		Biotin--acetyl-CoA-carboxylase ligase	795	649293	1178005	856704	0,5038	0.4149+-0.0112	0.5971+-0.0317	✓	✓	✓
bhsal07770		Uncharacterized protein	912	833649	1369640	928269	0,4836	0.4265+-0.0197	0.5506+-0.0243	✓	✓	✓
bhsal05400	*rplF*	50S ribosomal protein L6	531	568265	1477425	975788	0,4859	0.4660+-0.0090	0.5042+-0.0109	✓	✓	
bhsal12030	*hslU*	ATP-dependent protease ATPase subunit HslU	1305	1317133	312957	157433	0,5023	0.4840+-0.0042	0.5220+-0.0137	✓		
bhsal09160		Alkyl hydroperoxide reductase	483	985199	1199456	834729	0,4784	0.4333+-0.0161	0.5257+-0.0252	✓	✓	
bhsal16640	*grpE*	Protein GrpE	690	1820646	77441	75104	0,5325	0.4914+-0.0167	0.5716+-0.0192	✓	✓	
bhsal09040	*rlpA*	Rare lipoprotein A	729	970709	1229825	807358	0,4898	0.4074+-0.0206	0.5725+-0.0256	✓	✓	✓
bhsal08890		Uncharacterized protein	4662	957509	1298116	735879	0,4955	0.4192+-0.0137	0.5740+-0.0064	✓	✓	✓
bhsal11680		Uncharacterized protein	276	1263633	659613	1118702	0,5161	0.4574+-0.0284	0.5698+-0.0332	✓	✓	
bhsal09120		Chromosome I- genome	672	978431	1201892	831995	0,4956	0.4744+-0.0066	0.5157+-0.0087	✓		
bhsal07590		Uncharacterized protein	510	810477	1286259	746106	0,4591	0.3988+-0.0156	0.5148+-0.0211	✓	✓	✓
bhsal09810		Uncharacterized protein	237	1058340	1341740	903122	0,5125	0.4833+-0.0192	0.5437+-0.0122			
**Total**										39	26	14

[a] Canonical Bartonella spp. are monophyletic

[b] Canonical Bartonella spp. are monophyletic, and are subtended with B. tamiae

[c] Canonical Bartonella spp. are monophyletic, are subtended with B. tamiae, and this group is subtended by Bhsal

Supplementary Table S4 List of ancestrally acquired genes. The list includes genes inferred as gained in the common ancestor of Bhsal, *Bartonella* and *B. tamiae*.

#	Sequences	COG category	COG	Seqs classified	Locus tag in BG (if gene present) or others		annotation
1	18	R	COG1783 Phage terminase large subunit	4	BGr07730		phage terminase, large subunit
					BGr09810		phage terminase, large subunit
					BGr16090		phage terminase, large subunit
2	18	U	COG3210 Large exoproteins involved in heme utilization or adhesion	12	BGr07650	*fhaB2*	filamentous hemagglutinin
					BGr07860	*fhaB3*	filamentous hemagglutinin
3	17	ET	COG0834 ABC-type amino acid transport/signal transduction systems, periplasmic component/domain	7	BGr12890	*gltI*	glutamate-aspartate ABC transporter periplasmic component
4	17	-	Not classified	0	BGr07740		putative phage portal protein
					BGr09820		putative phage portal protein
					BGr16080		putative phage portal protein
5	11	S	COG3827 Uncharacterized protein conserved in bacteria	7	BGr12960		hypothetical protein
6	11	P	COG0619 ABC-type cobalt transport system, permease component CbiQ and related transporters	11	BGr02910		putative biotin ABC transporter, permease protein
7	11	P	COG1122 ABC-type cobalt transport system, ATPase component	11	BGr02900		ABC transporter ATP-binding protein
8	11	C	COG1301 Na+/H+-dicarboxylate symporters	2	BGr04200		sodium/dicarboxylate symporter
		R	COG1823 Predicted Na+/dicarboxylate symporter	9			

9	12	C	COG0584 Glycerophosphoryl diester phosphodiesterase	12	BGr01870	*glpQ*	glycerophosphoryl diester phosphodiesterase
10	11	E	COG0345 Pyrroline-5-carboxylate reductase	11	BGr09160	*proC*	pyrroline-5-carboxylate reductase
11	10	U	COG2831 Hemolysin activation/sec-retion protein	10	BGr07640	*hec2*	Hemolysin activator protein Hec
					BGr07850	*hec3*	Hemolysin activator protein Hec
12	9	-	Not classified	0	BGr13000		hypothetical protein
13	9	-	Not classified	0	BGr04280		hypothetical protein
14	9	E	COG0531 Amino acid transporters	1	BGr12140		amino acid permease
		E	COG1113 Gamma-aminobutyrate permease and related permeases	8			
15	9	M	COG3637 Opacity protein and related surface antigens	9	BGr05090	*hbp5*	hemin binding protein
16	8	-	Not classified	0	BGr12020		hypothetical protein
17	8	-	Not classified	0	BGr16060		phage related protein
18	9	-	Not classified	0	BGr16070		hypothetical protein
19	9	-	Not classified	0	BGr16100		hypothetical protein
20	7	S	COG1652 Uncharacterized protein containing LysM domain	7	BGr00620		LysM domain/BON superfamily protein
21	8	-	Not classified	0	m07a09730		hypothetical protein
22	7	-	Not classified	0	BGr15950		hypothetical protein
23	5	R	COG1853 Conserved protein/domain typically associated with flavoprotein oxygenases, DIM6/NTAB family	1	BJaplr3398		hypothetical protein
24	3	H	COG1893 Ketopantoate reductase	3	bhsal04060		2-dehydropantoate 2-reductase
25	3	K	COG0846 NAD-dependent protein deacetylases, SIR2 family	3	bhsal13150	*cobB*	NAD-dependent protein deacylase

26	3	V	COG2274 ABC-type bacteriocin/lantibiotic exporters, contain an N-terminal double-glycine peptidase domain	2	BJaplr5468		ABC transporter
		R	COG4618 ABC-type protease/lipase transport system, ATPase and permease components	1			
27	3	-	Not classified	0	BGr06170		hypothetical protein
28	2	-	Not classified	0	bhsal08030		Unique protein
29	2	-	Not classified	0	bhsal11970		Unique protein
30	2	P	COG0725 ABC-type molybdate transport system, periplasmic component	2	BJaplr6951	*modA*	molybdenum ABC transporter molybdate-binding protein
31	2	-	Not classified	0	BJaplr0239		citrate-proton symporter
32	2	-	Not classified	0	bhsal07760		Uncharacterized protein
33	2	S	COG3162 Predicted membrane protein	2	bhsal03970		Uncharacterized protein
34	2	R	COG4147 Predicted symporter	2	bhsal03980	*actP*	Actetate permease
35	2	G	COG1105 Fructose-1-phosphate kinase and related fructose-6-phosphate kinase (PfkB)	2	bhsal08000		Phosphofructokinase
36	2	M	COG1292 Choline-glycine betaine transporter	2	SM_b20333		transporter protein
37	1	E	COG0531 Amino acid transporters	1	bhsal00780		Amino acid permease
38	1	-	Not classified	0	bhsal13160		Unique protein
39	1	S	COG3477 Predicted periplasmic/secreted protein	1	bhsal01950		Uncharacterized protein
40	1	-	Not classified	0	bhsal00550		Signal peptide protein
41	1	-	Not classified	0	bhsal00690		Unique protein
42	1	-	Not classified	0	bhsal00680		Unique protein

43	1	R	COG5342 Invasion protein B, involved in pathogenesis	1	bhsal16380	Invasion associated locus B family protein
44	1	-	Not classified	0	bhsal16280	Unique protein
45	1	C	COG0843 Heme/copper-type cytochrome/quinol oxidases, subunit 1	1	bhsal13840	Unique protein
46	1	E	COG0079 Histidinol-phosphate/aromatic aminotransferase and cobyric acid decarboxylase	1	bhsal12390	Aminotransferase class I and II
47	1	S	COG4392 Predicted membrane protein	1	bhsal06530	Uncharacterized protein
48	1	-	Not classified	0	bhsal08250	Uncharacterized protein
49	1	-	Not classified	0	bhsal09660	Uncharacterized protein
50	1	-	Not classified	0	bhsal10250	Dihydroneopterin aldolase
51	1	-	Not classified	0	bhsal12430	Unique protein
52	1	-	Not classified	0	bhsal11960	Tryptophan halogenase
53	1	-	Not classified	0	bhsal03150	Uncharacterized protein
54	1	K	COG1475 Predicted transcriptional regulators	1	bhsal05070	Uncharacterized protein

Supplementary table S5 List of genes acquired in the ancestor of *Bartonella.*

#	Sequences	COG category	COG	Seqs classified	Locus tag in BG	Gene	Annotation
1	32	MU	COG3468 Type V secretory pathway, adhesin AidA	26	BG12090		autotransporter
					BG15480		autotransporter
					BG15490		autotransporter
					BG15500		autotransporter
2	28	MU	COG3468 Type V secretory pathway, adhesin AidA	17	BG09460	*iba1*	Inducible Bartonella autotransporter
					BG14050	*iba2*	Inducible Bartonella autotransporter
					BG14060	*iba3*	Inducible Bartonella autotransporter
					BG14070	*iba4*	Inducible Bartonella autotransporter
					BG14080	*iba5*	Inducible Bartonella autotransporter
					BG14100	*iba7*	Inducible Bartonella autotransporter
3	21	K	COG3561 Phage anti-repressor protein	21	BG03400		anti-repressor protein
					BG08740		anti-repressor protein
					BG09620		anti-repressor protein
4	19	U	COG2948 Type IV secretory pathway, VirB10 components	19	BG14390	*virB10*	type IV secretion system protein VirB10
					BG17370	*vblB10*	type IV secretion protein VblB10
					BGpBGR300150	*vblB10*	type IV secretion protein VblB10
5	18	U	COG3451 Type IV secretory pathway, VirB4 components	18	BG14330	*virB4*	type IV secretion system protein VirB4
					BG17270	*vblB4*	type IV secretion protein VblB4
					BGpBGR300080	*vblB4*	type IV secretion protein VblB4
6	18	U	COG3504 Type IV secretory pathway, VirB9 components	18	BG14380	*virB9*	type IV secretion system protein VirB9
					BG17360	*vblB9*	type IV secretion protein VblB9

					BGpBGR300140	*vblB9*	type IV secretion protein VblB9
7	18	U	COG3736 Type IV secretory pathway, component VirB8	18	BG14370	*virB8*	type IV secretion system protein VirB8
					BG17350	*vblB8*	type IV secretion protein VblB8
					BGpBGR300130	*vblB8*	type IV secretion protein VblB8
8	18	U	COG3704 Type IV secretory pathway, VirB6 components	18	BG14350	*virB6*	type IV secretion system protein VirB6
					BG17330	*vblB6*	type IV secretion protein VblB6
					BGpBGR300110	*vblB6*	type IV secretion protein VblB6
9	18	NU	COG0630 Type IV secretory pathway, VirB11 components, and related ATPases involved in archaeal flagella biosynthesis	17	BG14400	*virB11*	type IV secretion system protein VirB11
		U	COG4962 Flp pilus assembly protein, ATPase CpaF	1	BG17380	*vblB11*	type IV secretion protein VblB11
					BGpBGR300160	*vblB11*	type IV secretion protein VblB11
10	18	R	COG3895 Predicted periplasmic protein	17	BG19270		hypothetical protein
					BG19280		hypothetical protein
11	17	U	COG3702 Type IV secretory pathway, VirB3 components	17	BG14320	*virB3*	type IV secretion system protein VirB3
					BG17260	*vblB3*	type IV secretion protein VblB3
					BGpBGR300070	*vblB3*	type IV secretion protein VblB3
12	16	-	Not classified	0	BG14820		hypothetical membrane protein
					BG15840		hypothetical membrane protein
13	16	G	COG1653 ABC-type sugar transport system, periplasmic component	10	BG01880	*ugpB*	sn-glycerol 3-phosphate transport system substrate-binding protein
14	15	P	COG1283 Na+/phosphate symporter	15	BG13790		Na/Pi-cotransporter family protein
15	15	E	COG0683 ABC-type branched-chain amino acid transport systems, periplasmic component	15	BG10710	*livJ*	branched chain amino acid ABC transporter periplasmic ligand-binding protein

16	15				BG01890	*ugpA*	sn-glycerol 3-phosphate transport system permease protein
17	15				BG01900	*ugpE*	sn-glycerol 3-phosphate transport system permease protein
18	15	H	COG0414 Panthothenate synthetase	15	BG05820	*panC*	pantoate--beta-alanine ligase
19	15	V	COG1566 Multidrug resistance efflux pump	15	BG14950	*vceA*	multidrug resistance protein vceA
20	14	S	COG1806 Uncharacterized protein conserved in bacteria	14	BG00010		hypothetical protein
21	14	H	COG0413 Ketopantoate hydroxymethyltransferase	14	BG05830	*panB*	3-methyl-2-oxobutanoate hydroxymethyltransferase
22	14				BG14960	*vceB*	multidrug resistance protein vceB
23	13	L	COG1525 Micrococcal nuclease (thermonuclease) homologs	12	BG14300		putative nuclease
					BG17240		nuclease (SNase-like)
24	13	-	Not classified	0	BG03660		phage related protein
					BG07490		phage related protein
					BG08980		phage related protein
25	13	U	COG3505 Type IV secretory pathway, VirD4 components	13	BG14430	*virD4*	conjugal transfer protein TraG/VirD4
					BG16640	*traG2*	conjugal transfer protein TraG/VirD4
					BGpBGR300230	*traG*	conjugal transfer protein TraG/VirD3
26	13	-	Not classified	0	BG15820		multidrug resistance transporter, Bcr/CflA family
27	12	MU	COG3468 Type V secretory pathway, adhesin AidA	4	BG06200		pertactin family virulence factor/autotransporter
					BG06220		pertactin family virulence factor/autotransporter
28	12	S	COG5457 Uncharacterized conserved small protein	12	BG18860		hypothetical protein

29	12	H	COG0422 Thiamine biosynthesis protein ThiC	12	BG05600	*thiC*	thiamine biosynthesis protein ThiC
30	11	R	COG3772 Phage-related lysozyme (muraminidase)	10	BG03180		phage related lysozyme
					BG08410		phage related lysozyme
31	11	-	Not classified	0	BG03500		hypothetical protein
					BG08850		hypothetical protein
32	10	S	COG3646 Uncharacterized phage-encoded protein	5	BG03720		anti-repressor protein
					BG09040		anti-repressor protein
33	9	-	Not classified	0	BG03390		hypothetical protein
					BG08730		hypothetical protein
34	9	L	COG3077 DNA-damage-inducible protein J	9	BG10000	*dinJ*	DNA-damage-inducible protein J
35	9	-	Not classified	0	BG07450		hypothetical protein
36	9	-	Not classified	0	BG09390	*dppA*	ABC dipeptide transporter, substrate-binding subunit DppA
37	9	-	Not classified	0	BG17030		hypothetical protein
38	9	-	Not classified	0	BG16720		phage related protein
39	8	MU	COG3468 Type V secretory pathway, adhesin AidA	2	BG06210		pertactin family virulence factor/autotransporter
40	8	-	Not classified	0	BG03430		phage protein
					BG08780		phage protein
41	8	K	COG2002 Regulators of stationary/sporulation gene expression	3	BGpBGR300010		putative transcriptional regulator
42	8	R	COG5611 Predicted nucleic-acid-binding protein, contains PIN domain	1	BGpBGR300020		hypothetical protein
43	8	-	Not classified	0	BG13680		hypothetical DNA-binding protein
44	8	-	Not classified	0	BG15970		hypothetical protein
45	8	-	Not classified	0	BG16000		hypothetical protein
46	8	-	Not classified	0	BG16020		hypothetical membrane protein

47	8	-	Not classified	0	BG16030		hypothetical protein
48	8	-	Not classified	0	BG16040		hypothetical protein
49	8	-	Not classified	0	BG16050		hypothetical protein
50	8	-	Not classified	0	BG16680	*exo*	phage-related exonuclease
51	8	-	Not classified	0	BG16700		hypothetical protein
52	8	-	Not classified	0	BG16710		hypothetical protein
53	7	-	Not classified	0	BG08160	*yacA*	YacA protein
					BG14610	*yacA*	YacA protein
54	7	S	COG3041 Uncharacterized protein conserved in bacteria	7	BG09990		addiction module toxin, RelE/StbE family
55	7	-	Not classified	0	BG06180		hypothetical protein
56	7	-	Not classified	0	BG13690		hypothetical protein
57	7	H	COG3161 4-hydroxybenzoate synthetase (chorismate lyase)	7	BG13810	*ubiC*	chorismate--pyruvate lyase
58	7	-	Not classified	0	BG16690		hypothetical protein

Table S6 List of genes acquired in the ancestor of Bhsal.

#	Sequences	COG category	COG	Seqs classified	Locus tag in Gene Bhsal	Annotation
1	11	MU	COG3468 Type V secretory pathway, adhesin AidA	10	bhsal03490	Unique protein
					bhsal15270	Outer membrane autotransporter barrel domain-containing protein (Precursor)
2	12	-	Not classified	0	bhsal09530	Transporter, major facilitator family
3	11	R	COG3093 Plasmid maintenance system antidote protein	9	bhsal16290	Putative plasmid maintenance system antidote protein, XRE family
4	9	-	Not classified	0	bhsal06590	Unique protein
					bhsal06600	Unique protein
5	8	-	Not classified	0	bhsal16040	Uncharacterized protein
6	8	-	Not classified	0	bhsal01600	Transcriptional regulator (Precursor)
7	6	-	Not classified	0	bhsal00740	Lytic transglycosylase
8	7	R	COG1708 Predicted nucleotidyltransferases	7	bhsal03010	Nucleotidyltransferase
					bhsal05650	Putative nucleotidyltransferase
					bhsal06800	Nucleotidyltransferase
					bhsal16390	Putative nucleotidyltransferase
9	7	P	COG2608 Copper chaperone	7	bhsal10890	Heavy-metal-associated domain-containing protein
10	6				bhsal00810	Hypothetical protein
11	6	P	COG2223 Nitrate/nitrite transporter	6	bhsal10310	Uncharacterized protein
					bhsal10320	Nitrite extrusion protein
12	5	O	COG2370 Hydrogenase/urease accessory protein	5	bhsal04990	Urease accessory protein

13	5	I	COG1182 Acyl carrier protein phosphodiesterase	5	bhsal01590	*azoR*	FMN-dependent NADH-azoreductase
14	5	ET	COG0834 ABC-type amino acid transport/signal transduction systems, periplasmic component/domain	1	bhsal10550		Amino acid ABC transporter substrate-binding protein
15	5	P	COG2239 Mg/Co/Ni transporter MgtE (contains CBS domain)	5	bhsal06840		Magnesium transporter
16	4	K	COG2944 Predicted transcriptional regulator	4	bhsal16320		Putative DNA-binding protein
17	5				bhsal11560		Putative peptidase M38 family protein
18	5	R	COG2103 Predicted sugar phosphate isomerase	5	bhsal11290	*murQ*	N-acetylmuramic acid 6-phosphate etherase
19	4	-	Not classified	0	bhsal02980		Uncharacterized protein
					bhsal16420		Uncharacterized protein
20	4	M	COG0463 Glycosyltransferases involved in cell wall biogenesis	1	bhsal16740		Unique protein
					bhsal16750		Uncharacterized protein
21	4	H	COG0156 7-keto-8-aminopelargonate synthetase and related enzymes	1	bhsal03080		2-amino-3-ketobutyrate coenzyme A ligase
22	4	S	COG3152 Predicted membrane protein	4	bhsal15770	*yhaI*	Putative membrane protein
23	4	V	COG2367 Beta-lactamase class A	4	bhsal05730		Beta-lactamase class A protein
24	4	S	COG4125 Predicted membrane protein	4	bhsal08870		Transmembrane pair
25	4	GER	COG0697 Permeases of the drug/metabolite transporter (DMT) superfamily	1	bhsal12040		Uncharacterized protein
26	4	K	COG1309 Transcriptional regulator	2	bhsal01370	*betI*	HTH-type transcriptional regulator BetI

27	4	I	COG1835 Predicted acyltransferases	4	bhsal09570		Unique protein
28	4	G	COG2271 Sugar phosphate permease	1	bhsal03100	*glpT*	sn-glycerol-3-phosphate transport protein (MFS family)
29	4	ER	COG1063 Threonine dehydrogenase and related Zn-dependent dehydrogenases	2	bhsal04440		Putative zinc-binding alcohol dehydrogenase
30	4	S	COG3600 Uncharacterized phage-associated protein	4	bhsal11210		Putative phage-associated protein
					bhsal12160	*gepA*	Phage-associated protein
31	4				bhsal02000		Unique protein
32	3	P	COG0471 Di- and tricarboxylate transporters	3	bhsal03300		Citrate:succinate antiporter
					bhsal06010		Divalent anion:Na+ symporter (DASS) family transporter
33	4	E	COG2195 Di- and tripeptidases	4	bhsal04330		Peptidase T
34	2	-	Not classified	0	bhsal00030		Type I secretion system outer membrane protein
					bhsal11940		Uncharacterized protein
35	3	S	COG3152 Predicted membrane protein	3	bhsal08140		Unique protein
					bhsal09410		Uncharacterized protein
36	3	E	COG0010 Arginase/agmatinase/formimionoglutamate hydrolase, arginase family	3	bhsal10720		Agmatinase
37	3	S	COG3791 Uncharacterized conserved protein	2	bhsal06050		Uncharacterized protein
38	3	OU	COG0740 Protease subunit of ATP-dependent Clp proteases	3	bhsal03510	*clpP*	ATP-dependent Clp protease proteolytic subunit

39	3	S	COG4625 Uncharacterized protein with a C-terminal OMP (outer membrane protein) domain	3	bhsal11570		Unique protein
40	3	E	COG0620 Methionine synthase II (cobalamin-independent)	3	bhsal11070	*metE*	5-methyltetrahydropteroyltriglutamate-- homocysteine methyltransferase
41	3	R	COG1540 Uncharacterized proteins, homologs of lactam utilization protein B	3	bhsal08600		UPF0271 protein USDA257_c17800
42	3	S	COG4336 Uncharacterized conserved protein	3	bhsal08610		UPF0317 protein PROVRUST_07488
43	3	E	COG2049 Allophanate hydrolase subunit 1	1	bhsal08620		Uncharacterized protein
44	3	-	Not classified	0	bhsal10530		Putative amino acid permease
45	3	S	COG5430 Uncharacterized secreted protein	3	bhsal11800		Unique protein
46	3	NU	COG3188 P pilus assembly protein, porin PapC	3	bhsal11810	*csuD*	Putative outer membrane usher protein yehB
47	3	-	Not classified	0	bhsal07070		Uncharacterized protein
48	3	-	Not classified	0	bhsal01510		Unique protein
49	3	E	COG2856 Predicted Zn peptidase	1	bhsal08170		Uncharacterized protein
50	3	R	COG1380 Putative effector of murein hydrolase LrgA	3	bhsal02060		LrgA family protein
51	3	-	Not classified	0	bhsal07920		Zinc-containing alcohol dehydrogenase superfamily
52	3	-	Not classified	0	bhsal10190		Major facilitator family transporter
53	3	E	COG1115 Na+/alanine symporter	3	bhsal12410	*glnT*	Putative sodium/glutamine symporter GlnT
54	2	-	Not classified	0	bhsal16310	*relE*	RelE protein
55	3	-	Not classified	0	bhsal00760		Hypothetical protein

56	3	-	Not classified	0	bhsal09450		Uncharacterized protein
57	3	E	COG0549 Carbamate kinase	3	bhsal10220		Carbamate kinase
58	3	E	COG0160 4-aminobutyrate aminotransferase and related aminotransferases	3	bhsal10700		4-aminobutyrate aminotransferase
59	2	-	Not classified	0	bhsal02990		Putative uncharacterized protein
					bhsal16410		Putative uncharacterized protein
60	2	FR	COG0402 Cytosine deaminase and related metal-dependent hydrolases	2	bhsal09710		Putative deaminase with metallo-dependent hydrolase domain
					bhsal10160		Putative deaminase with metallo-dependent hydrolase domain
61	2	-	Not classified	0	bhsal00380		Major facilitator family transporter
					bhsal00390		Major facilitator family transporter
62	2	R	COG3772 Phage-related lysozyme (muraminidase)	1	bhsal00800		Unique protein
					bhsal00840		Phage related lysozyme
63	2	-	Not classified	0	bhsal02970		Unique protein
					bhsal16430		Unique protein
64	2	-	Not classified	0	bhsal03520		Unique protein
					bhsal03530		Unique protein
65	2	-	Not classified	0	bhsal06990		Unique protein
					bhsal09260		Unique protein
66	2	-	Not classified	0	bhsal13690		Unique protein
					bhsal13700		Unique protein
67	2	S	COG4922 Uncharacterized protein conserved in bacteria	2	bhsal15760		Uncharacterized protein
68	2	S	COG3251 Uncharacterized protein conserved in bacteria	2	bhsal07080	*mbtH*	Protein mbtH
69	2	P	COG1914 Mn2+ and Fe2+ transporters of the NRAMP family	2	bhsal08590		Membrane protein, putative

70	2	I	COG4770 Acetyl/propionyl-CoA carboxylase, alpha subunit	2	bhsal08630		Acetyl/propionyl-CoA carboxylase, alpha subunit
71	2	M	COG0562 UDP-galactopyranose mutase	2	bhsal16760	*glf*	UDP-galactopyranose mutase
72	2	P	COG1513 Cyanate lyase	2	bhsal11280	*cynS*	Cyanate hydratase
73	2	MG	COG0451 Nucleoside-diphosphate-sugar epimerases	2	bhsal03090		Uncharacterized protein
74	2	-	Not classified	0	bhsal01670		Uncharacterized protein
75	2	I	COG0671 Membrane-associated phospholipid phosphatase	2	bhsal09050		Acid phosphatase
76	2	ER	COG1063 Threonine dehydrogenase and related Zn-dependent dehydrogenases	2	bhsal04190	*adh*	Alcohol dehydrogenase Adh
77	2	-	Not classified	0	bhsal05700		Uncharacterized protein (Precursor)
78	2	-	Not classified	0	bhsal05710		Putative oxidoreductase
79	2	-	Not classified	0	bhsal16770		Unique protein
80	1	-	Not classified	0	bhsal00250		Unique protein
81	1	-	Not classified	0	bhsal00260		Unique protein
82	1	E	COG0665 Glycine/D-amino acid oxidases (deaminating)	1	bhsal00350		FAD-dependent oxidoreductase
83	1	-	Not classified	0	bhsal00750		Hypothetical periplasmic protein
84	1	-	Not classified	0	bhsal00790		Hypothetical protein
85	1	-	Not classified	0	bhsal00850		Unique protein
86	1	-	Not classified	0	bhsal01100		Unique protein
87	1	E	COG1168 Bifunctional PLP-dependent enzyme with beta-cystathionase and maltose regulon repressor activities	1	bhsal01160		Aspartate aminotransferase
88	1	-	Not classified	0	bhsal02320		Unique protein
89	1	-	Not classified	0	bhsal02380		Unique protein

90	1	-	Not classified	0	bhsal02780		Unique protein
91	1	V	COG1002 Type II restriction enzyme, methylase subunits	1	bhsal02840		Putative DNA methylase
92	1	-	Not classified	0	bhsal02850		Pseudomurein-binding repeat protein
93	1	-	Not classified	0	bhsal02860		Putative uncharacterized protein
94	1	-	Not classified	0	bhsal03000		Putative uncharacterized protein
95	1	-	Not classified	0	bhsal03240		Unique protein
96	1	-	Not classified	0	bhsal03600		Unique protein
97	1	-	Not classified	0	bhsal04050		Unique protein
98	1	-	Not classified	0	bhsal04170		Unique protein
99	1	-	Not classified	0	bhsal04220		Unique protein
100	1	-	Not classified	0	bhsal04230		2,4-diacetylphloroglucinol specific hydrolase PhlG
101	1	E	COG1126 ABC-type polar amino acid transport system, ATPase component	1	bhsal04350		General amino acid ABC transporter, ATP-binding protein
102	1	-	Not classified	0	bhsal04380	*argT*	Lysine-arginine-ornithine-binding periplasmic protein ArgT
103	1	R	COG1473 Metal-dependent amidase/aminoacylase/carboxypeptidase	1	bhsal04470		Amidohydrolase
104	1	-	Not classified	0	bhsal04570		Putative invasin, attaching and effacing protein
105	1	-	Not classified	0	bhsal05080		Unique protein
106	1	R	COG3969 Predicted phosphoadenosine phosphosulfate sulfotransferase	1	bhsal05090		Phosphoadenosine phosphosulfate reductase family protein
107	1	-	Not classified	0	bhsal05520		Glucose-1-phosphatase
108	1	O	COG0501 Zn-dependent protease with chaperone function	1	bhsal05640		Uncharacterized protein
109	1	-	Not classified	0	bhsal05660		Uncharacterized protein

110	1	R	COG1203 Predicted helicases	1	bhsal05740	*cas3*	CRISPR-associated helicase, Cas3 family
111	1	-	Not classified	0	bhsal05750		Unique protein
112	1	-	Not classified	0	bhsal05760	*cas5*	CRISPR-associated protein Cas5
113	1	-	Not classified	0	bhsal05770		CRISPR-associated protein, Csd1 family
114	1	L	COG3649 Uncharacterized protein predicted to be involved in DNA repair	1	bhsal05780		CRISPR-associated protein, Csd2 family
115	1	L	COG1468 RecB family exonuclease	1	bhsal05790	*cas4*	CRISPR-associated protein Cas4
116	1	L	COG1518 Uncharacterized protein predicted to be involved in DNA repair	1	bhsal05800	*cas1*	CRISPR-associated endonuclease Cas1
117	1	L	COG1343 Uncharacterized protein predicted to be involved in DNA repair	1	bhsal05810	*cas2*	CRISPR-associated endoribonuclease Cas2
118	1	-	Not classified	0	bhsal05870		Unique protein
119	1	-	Not classified	0	bhsal05950		Unique protein
120	1	S	COG3513 Uncharacterized protein conserved in bacteria	1	bhsal06180	*cas9*	CRISPR-associated protein
121	1	L	COG1518 Uncharacterized protein predicted to be involved in DNA repair	1	bhsal06190	*cas1*	CRISPR-associated endonuclease Cas1
122	1	S	COG3512 Uncharacterized protein conserved in bacteria	1	bhsal06200	*cas2*	CRISPR-associated endoribonuclease Cas2 (Precursor)
123	1	-	Not classified	0	bhsal06240		Uncharacterized protein
124	1	-	Not classified	0	bhsal06300		Unique protein
125	1	-	Not classified	0	bhsal06570		Unique protein
126	1	-	Not classified	0	bhsal06580		Unique protein
127	1	-	Not classified	0	bhsal06610		Uncharacterized protein
128	1	-	Not classified	0	bhsal06680		Unique protein

129	1	-	Not classified	0	bhsal07110		Phosphorylcholine phosphatase
130	1	-	Not classified	0	bhsal07230		Uncharacterized protein
131	1	-	Not classified	0	bhsal07660		Unique protein
132	1	-	Not classified	0	bhsal07810		Unique protein
133	1	-	Not classified	0	bhsal07830		Unique protein
134	1	-	Not classified	0	bhsal08150		Unique protein
135	1	-	Not classified	0	bhsal08160		Unique protein
136	1	-	Not classified	0	bhsal08190	*mucR*	Transcriptional regulatory protein MucR
137	1	-	Not classified	0	bhsal08490		Major facilitator superfamily MFS_1
138	1	-	Not classified	0	bhsal08760		Unique protein
139	1	-	Not classified	0	bhsal08970		Uncharacterized protein
140	1	-	Not classified	0	bhsal09170		Unique protein
141	1	-	Not classified	0	bhsal09420		Unique protein
142	1	-	Not classified	0	bhsal09650		Unique protein
143	1	-	Not classified	0	bhsal09740		Unique protein
144	1	R	COG0666 FOG: Ankyrin repeat	1	bhsal09750		Ankyrin repeat protein
145	1	R	COG1473 Metal-dependent amidase/aminoacylase/carboxypeptidase	1	bhsal09840		Amidohydrolase
146	1	-	Not classified	0	bhsal10050		Unique protein
147	1	-	Not classified	0	bhsal10060		Unique protein
148	1	-	Not classified	0	bhsal10150		Unique protein
149	1	-	Not classified	0	bhsal10180		UIT9 transporter
150	1	-	Not classified	0	bhsal10200		Uncharacterized protein
151	1	C	COG0074 Succinyl-CoA synthetase, alpha subunit	1	bhsal10210		Uncharacterized protein
152	1	G	COG1263 Phosphotransferase system IIC components, glucose/maltose/N-acetylglucosamine-specific	1	bhsal10420	*nagE*	Protein-N p-phosphohistidine-sugar phosphotransferase

153	1	-	Not classified	0	bhsal10430		Unique protein
154	1	-	Not classified	0	bhsal10440		Nuclease-related domain protein
155	1	-	Not classified	0	bhsal11060		Unique protein
156	1	-	Not classified	0	bhsal11220		Unique protein
157	1	P	COG0288 Carbonic anhydrase	1	bhsal11270		Carbonic anhydrase
158	1	-	Not classified	0	bhsal11410		Unique protein
159	1	-	Not classified	0	bhsal11450		Unique protein
160	1	R	COG3568 Metal-dependent hydrolase	1	bhsal11910		Uncharacterized protein
161	1	-	Not classified	0	bhsal11950		Unique protein
162	1	-	Not classified	0	bhsal12170		Unique protein
163	1	-	Not classified	0	bhsal12350		Unique protein
164	1	-	Not classified	0	bhsal12380		Unique protein
165	1	-	Not classified	0	bhsal12570		Unique protein
166	1	-	Not classified	0	bhsal12950		Uncharacterized protein
167	1	-	Not classified	0	bhsal13420		Unique protein
168	1	-	Not classified	0	bhsal13560		Unique protein
169	1	T	COG1966 Carbon starvation protein, predicted membrane protein	1	bhsal13570		Carbon starvation protein A
170	1	S	COG2879 Uncharacterized small protein	1	bhsal13580		Uncharacterized protein
171	1	-	Not classified	0	bhsal13610		Unique protein
172	1	C	COG1902 NADH:flavin oxidoreductases, Old Yellow Enzyme family	1	bhsal13930		NADH:flavin oxidoreductase/NADH oxidase
173	1	E	COG0814 Amino acid permeases	1	bhsal14140	*sdaC*	Serine transporter
174	1	-	Not classified	0	bhsal14390		Unique protein
175	1	-	Not classified	0	bhsal14600		Unique protein
176	1	-	Not classified	0	bhsal14650		Unique protein
177	1	-	Not classified	0	bhsal14670		Unique protein

178	1	-	Not classified	0	bhsal15220		Uncharacterized protein
179	1	-	Not classified	0	bhsal15410	*doxX*	Uncharacterized protein
180	1	E	COG0665 Glycine/D-amino acid oxidases (deaminating)	1	bhsal15780		FAD dependent oxidoreductase
181	1	-	Not classified	0	bhsal16050		Unique protein
182	1	-	Not classified	0	bhsal16070		Unique protein
183	1	-	Not classified	0	bhsal16200		Unique protein
184	1	L	COG3316 Transposase and inactivated derivatives	1	bhsal16300		Uncharacterized protein
185	1	-	Not classified	0	bhsal16350		Uncharacterized protein
186	1	-	Not classified	0	bhsal16400		Unique protein
187	1	-	Not classified	0	bhsal16480		Unique protein
188	1	-	Not classified	0	bhsal16940		Unique protein
189	1	-	Not classified	0	bhsal16970		Uncharacterized protein

Supplementary table S7 List of genes lost in the ancestor of Bhsal.

#	Sequences	COG category	COG	Seqs classified	Locus tag in *B. grahamii* (alt. *B. shoebuchensis*)	Gene	Annotation
1	29	-	Not classified	0	BGr09290		ABC transporter, periplasmic oligopeptide-binding protein
					BGr09310		ABC transporter, periplasmic oligopeptide-binding protein
					BGr09330		ABC transporter, periplasmic oligopeptide-binding protein
					BGr09380		ABC transporter, periplasmic oligopeptide-binding protein
2	29	-	Not classified	0	BGr03140	*int*	phage integrase
					BGr07680	*int*	phage integrase
					BGr07960	*int*	phage integrase
					BGr08190	*int*	phage integrase
					BGr08380	*int*	phage integrase
					BGr14580	*int*	phage integrase
					BGr15290	*int*	phage integrase
					BGr16420	*int*	phage integrase
3	25	EP	COG1124 ABC-type dipeptide/oligopeptide/nickel transport system, ATPase component	15	BGr09430	*dppF*	dipeptide ABC transporter, ATP-binding protein
		E	COG4608 ABC-type oligopeptide transport system, ATPase component	10			

4	26	EP	COG0444 ABC-type dipeptide/oligopeptide/nickel transport system, ATPase component	11	BGr09420	*dppD*	dipeptide ABC transporter, ATP-binding protein
5	19	S	COG4782 Uncharacterized protein conserved in bacteria	19	BGr10990		hypothetical protein
6	19				BGr05210		hypothetical protein
7	19	K	COG1278 Cold shock proteins	15	BGr05480	*cspA*	cold shock protein (beta-ribbon, CspA family)
8	18	V	COG0841 Cation/multidrug efflux pump	12	BGr01330	*acrD*	acriflavin resistance protein D
9	16	E	COG0410 ABC-type branched-chain amino acid transport systems, ATPase component	16	BGr10750	*livF*	branched-chain amino acid transport system ATP-binding protein
10	15	C	COG3474 Cytochrome c2	15	BGr02410	*cyc*	cytochrome c
11	15				BGr01910	*ugpC*	sn-glycerol 3-phosphate transport system ATP-binding protein
12	14	K	COG3311 Predicted transcriptional regulator	14	BGr07950		hypothetical protein
					BGr14590		hypothetical DNA-binding protein
					BGr15280		hypothetical DNA-binding protein
					BGr16410		hypothetical DNA-binding protein
13	14	D	COG0424 Nucleotide-binding protein implicated in inhibition of septum formation	14	BGr00020	*maf1*	septum formation maf protein
14	14	E	COG0169 Shikimate 5-dehydrogenase	14	BGr00030	*aroE*	shikimate 5-dehydrogenase
15	14	H	COG0237 Dephospho-CoA kinase	14	BGr00040	*coaE*	dephospho-CoA kinase
16	14	L	COG0847 DNA polymerase III, epsilon subunit and related 3'-5' exonucleases	14	BGr00050	*dnaQ*	DNA polymerase III subunit epsilon

17	14	L	COG0749 DNA polymerase I - 3'-5' exonuclease and polymerase domains	14	BGr00060	*polA*	DNA polymerase I
18	14	L	COG0249 Mismatch repair ATPase (MutS family)	14	BGr00960	*mutS*	DNA mismatch repair protein MutS
19	14	-	Not classified	0	BGr04460		oxidoreductase
20	14	-	Not classified	0	BGr04990	*feuP*	two-component system regulatory protein
21	14	-	Not classified	0	BGr05000	*feuQ*	sensor histidine kinase
22	14	H	COG1995 Pyridoxal phosphate biosynthesis protein	14	BGr06110	*pdxA*	4-hydroxythreonine-4-phosphate dehydrogenase
23	14	-	Not classified	0	BGr10790	*aidB*	acyl-CoA dehydrogenase
24	14	G	COG0120 Ribose 5-phosphate isomerase	14	BGr07090	*rpiA*	ribose 5-phosphate isomerase A
25	14	H	COG0854 Pyridoxal phosphate biosynthesis protein	14	BGr13140	*pdxJ*	pyridoxal phosphate biosynthetic protein PdxJ
26	14	-	Not classified	0	BGr01320		HlyD family secretion protein
27	14	L	COG0210 Superfamily I DNA and RNA helicases	14	BGr13340	*uvrD*	DNA helicase II
28	14	EF	COG0505 Carbamoylphosphate synthase small subunit	14	BGr13830	*carA*	carbamoyl-phosphate synthase small subunit
29	14	EF	COG0458 Carbamoylphosphate synthase large subunit (split gene in MJ)	14	BGr05490	*carB*	carbamoyl-phosphate synthase large subunit
30	14	H	COG2022 Uncharacterized enzyme of thiazole biosynthesis	14	BGr05570	*thiG*	thiamine biosynthesis ThiG
31	14	-	Not classified	0	BGr05590	*thiO*	glycine oxidase
32	14	R	COG0486 Predicted GTPase	14	BGr19580	*trmE*	tRNA modification GTPase
33	14	K	COG1158 Transcription termination factor	14	BGr19590	*rho*	transcription termination factor Rho

34	14	H	COG0352 Thiamine monophosphate synthase	14	BGr05560	*thiE*	thiamine-phosphate pyrophosphorylase ThiE
35	14	-	Not classified	0	BGr05370<br>BGr05390		hypothetical protein<br>hypothetical protein
36	13	-	Not classified	0	BGr06000		hypothetical protein
37	13	P	COG4558 ABC-type hemin transport system, periplasmic component	13	BGr05650	*hmuT*	hemin ABC transporter, periplasmic hemin-binding protein
38	13	H	COG2104 Sulfur transfer protein involved in thiamine biosynthesis	13	BGr05580	*thiS*	thiamin biosynthesis protein ThiS
39	13	E	COG1280 Putative threonine efflux protein	11	BGr00210		amino acid efflux protein, LysE family
40	13	EJ	COG0252 L-asparaginase/archaeal Glu-tRNAGln amidotransferase subunit D	13	BGr02110	*ansA*	L-asparaginase
41	13	S	COG2261 Predicted membrane protein	13	BGr13800		transglycosylase-associated protei
42	12	-	Not classified	0	BGr15270<br>BGr16400		virulence-associated protein E<br>virulence-associated protein E
43	12	J	COG1189 Predicted rRNA methylase	12	BGr04580	*tlyA*	hemolysin-like protein
44	12	I	COG0365 Acyl-coenzyme A synthetases/AMP-(fatty) acid ligases	12	BGr18210	*acs*	acetyl-CoA synthetase
45	11	O	COG0071 Molecular chaperone (small heat shock protein)	11	BGr00920	*ibpA1*	heat shock protein
46	11	S	COG1671 Uncharacterized protein conserved in bacteria	11	BGr19090		hypothetical protein
47	11	E	COG0460 Homoserine dehydrogenase	11	BGr11870	*hom*	homoserine dehydrogenase
48	11	M	COG3264 Small-conductance mechanosensitive channel	11	BGr07260		mechanosensitive ion channel family protein
49	11	R	COG3153 Predicted acetyltransferase	11	BGr14970		acetyltransferase

50	11	P	COG3720 Putative heme degradation protein	11	BGr05660	*hmuS*	hemin degrading protein
51	11	-	Not classified	0	BGr11830		hypothetical membrane protein
52	10	G	COG0364 Glucose-6-phosphate 1-dehydrogenase	10	BGr04110	*zwf*	glucose-6-phosphate 1-dehydrogenase
53	10	H	COG0351 Hydroxymethylpyrimidine/phosphomethylpyrimidine kinase	10	BGr19180	*thiD2*	phosphomethylpyrimidine kinase
54	9	EG	COG0129 Dihydroxyacid dehydratase/phosphogluconate dehydratase	9	BGr04130	*edd*	phosphogluconate dehydratase
55	9	L	COG0350 Methylated DNA-protein cysteine methyltransferase	6	BGr05510		methyltransferase (fragment)
56	9	G	COG0800 2-keto-3-deoxy-6-phosphogluconate aldolase	9	BGr15010	*eda*	2-dehydro-3-deoxyphosphogluconate aldolase / 4-hydroxy-2-oxoglutarate aldolase
57	9	P	COG2032 Cu/Zn superoxide dismutase	9	BGr10410	*sodC*	Cu/Zn superoxide dismutase
58	9	-	Not classified	0	BGr07270		hypothetical protein
59	9	-	Not classified	0	m07a03150		hypothetical protein

Supplementary Table S8. The Low-quality areas in Bhsal draft genome.

#	start	end	primer left	primer right	Length	Action	Original problem	Primary Annotation	Genome region*	Corrected annotation*
1	2084	2092	1849	2249	400	Use PCR from original gap closure	Shallow coverage	tRNA-Arg	1849..2270	
2	198988	198988	198677	200221	1544	Corrected consesus based on new PCR	No coverage	adenocylhomocysteinase	198677..200039	
3	402538	402545	402216	403397	1181	Corrected consesus based on new PCR	No coverage	iron compound ABC transporter; hypothetical protein	402233..403451	
4	467251	467350	466105	468453	2348	*Shotgun sequence product*	No coverage	NADH:flavin oxidoreductase; 2 WD-repeat containing protein; cobalamin synthase	466139..468484	WD-repeat containing protein; Cobalamin synthetase protein
5	488501	492700	488406	494427	6021	*Shotgun sequence product*	Repeat structure	attaching and effacing protein	488415..492753	Invasin
6	611901	629950	614429	625377	10948	Corrected consesus based on new PCR	No coverage	*Trichomonas vaginalis* repeat protein	612734..614422	CRISPR-Cas system spacers
7	659401	671300	665806	669099	3293	Corrected consesus based on new PCR	No coverage	*C.elegans* repeat protein	654850..658140	CRISPR-Cas system spacers
8	747084	747088	745154	747719	2565	Corrected consesus based on new PCR	Low coverage	seryl-tRNA synthetase; acid phosphatase SurE; protein-L-isoaspartate O-methyltransferase	730873..733412	
9	879483	879483	878279	879893	1614	Corrected consesus based on new PCR	No coverage	aspartyl-tRNA synthetase	863971..865586	
10	939801	939950	939643	943503	3860	Re-sequence	No coverage	urea amidolyase-like protein; hypothetical protein; biotin carboxylase; carbamoyl-phosphate synthase	925335..929199	Allophanate hydrolase; Acetyl/Propionyl-CoA carboxylase
11	1031051	1033100	1030774	1033614	2840	*Shotgun sequence product*	Collapsed repeats?	BadA; ialB	1016469..1019133	
12	1190748	1190753	1190914	1190914	365	Corrected consesus based on new PCR	No coverage	GTP-binding protein TypA/BipA	1176067..1176465	
13	1639589	1639601	1638391	1641074	2683	*Shotgun sequence product*	No coverage	ADP-ribose pyrophosphatase; Arginine biosynthesis bifunctional protein; peptidyl-prolyl cis-trans isomerase	1623921..1626608	
14	1735367	1735371	1733773	1736086	2313	Re-sequence	No coverage	Hypothetical proteins	1719279..1721405	Mitochodrial inner membrane protein; HemY domain protein
15	1756367	1756367	1756087	1757109	1022	Corrected consesus based on new PCR	No coverage	Hypothetical protein	1741405..1742428	Surface Antigen

* Location and annotation in Bhsal genome if it has changed
